# Knowledge Discovery from Biomedical Ontologies in Cross Domains

**DOI:** 10.1371/journal.pone.0160005

**Published:** 2016-08-22

**Authors:** Feichen Shen, Yugyung Lee

**Affiliations:** 1 Department of Health Sciences Research, Mayo Clinic, Rochester, Minnesota, United States of America; 2 School of Computing and Engineering, University of Missouri - Kansas City, Kansas City, Missouri, United States of America; Garvan Institute of Medical Research, AUSTRALIA

## Abstract

In recent years, there is an increasing demand for sharing and integration of medical data in biomedical research. In order to improve a health care system, it is required to support the integration of data by facilitating semantic interoperability systems and practices. Semantic interoperability is difficult to achieve in these systems as the conceptual models underlying datasets are not fully exploited. In this paper, we propose a semantic framework, called Medical Knowledge Discovery and Data Mining (MedKDD), that aims to build a topic hierarchy and serve the semantic interoperability between different ontologies. For the purpose, we fully focus on the discovery of semantic patterns about the association of relations in the heterogeneous information network representing different types of objects and relationships in multiple biological ontologies and the creation of a topic hierarchy through the analysis of the discovered patterns. These patterns are used to cluster heterogeneous information networks into a set of smaller topic graphs in a hierarchical manner and then to conduct cross domain knowledge discovery from the multiple biological ontologies. Thus, patterns made a greater contribution in the knowledge discovery across multiple ontologies. We have demonstrated the cross domain knowledge discovery in the MedKDD framework using a case study with 9 primary biological ontologies from Bio2RDF and compared it with the cross domain query processing approach, namely SLAP. We have confirmed the effectiveness of the MedKDD framework in knowledge discovery from multiple medical ontologies.

## Introduction

There is an increasing demand for sharing and integration of medical data in biomedical research. Heterogeneous information networking on the cloud are designed to enable compliant sharing of data based on the relationships across domains [1]. The Linked Open Data project is a notable effort for creating a knowledge space of RDF documents linked together and sharing a common ontology [2]. RDF is a metadata data model designed by the World Wide Web for conceptual modeling of information on the Web [3]. SPARQL Protocol and RDF Query Language is an RDF query language for semantic query language to retrieve data stored in RDF format [4]. According to the Linked Open Data project, the Web of Data currently consists of 4.7 billion RDF triples, which are interlinked by around 142 million RDF links (May 2009) [5]. Bio2RDF (Linked Data for the Life Sciences) [6] is one of the Linked Open Data projects in life science domains and has successfully converted bioinformatics databases such as *KEGG*, *DrugBank*, *MGI*, *HGNC* and several of NCBI databases into ontologies using Semantic Web technologies. Bio2RDF contains over 2.5 million triples and 0.19 million outlinks and 0.19 million inlinks [7].

In order to improve a health care system, it is required to conduct the integration of knowledge and data by facilitating medical ontologies and to support semantic interoperability systems and practices [8]. For the purpose, semantic interoperability is essential between heterogeneous ontologies and datasets [9]. The benefits of semantic interoperability are clear for improving accuracy and efficiency of diagnoses and treatment by sharing patient data and providing semantic-based criteria. However, integration and analysis of heterogeneous ontologies and datasets are a huge challenge in biomedical research since the mapping between datasets from different sources is not trivial [10]. For example, drug discovery research heavily relies on multiple information sources to validate potential drug candidates as shown in the Open PHACTS project [11].

In complicated domains, it not only takes time to develop and maintain ontologies [12], but it is also difficult to integrate relevant data that would be both practical and useful for biomedical research [13]. There have been various studies on using semantic techniques to improve data integration and share biomedical ontologies and datasets such as BioPortal [14], Bio2RDF [6] and OBO [15]. However, these efforts merely support physical integration of multiple biomedical ontologies without considering latent semantic relations of data. Furthermore, none of them has the ability to discover those semantic patterns in a systematic way. Semantic interoperability is difficult to achieve in these systems as the conceptual models underlying datasets are not fully exploited. In particular, human intervention is strongly required so that these are not suitable for comprehensive and accurate knowledge discovery especially from a large amount of data.

We need a systematic approach for more effective integration and analysis of ontologies [12]. In particular, we need innovative methodologies and applications for data integration and sharing [10]. This may be feasible through analysis of the heterogeneous information networks that represent different types of objects and links in cross domains [1]. In order to support dynamic processing of integrated cross domain data, a network-based data model such as resource description framework standards (RDF) and RDF Query Language (SPARQL) can be used for knowledge discovery from complex biomedical systems [16].

In this paper, we propose a semantic framework, called the Medical Knowledge Discovery and Data Mining (MedKDD), that aims to build a topic hierarchy and serve the semantic interoperability between different domains. In MedKDD, we fully focus on the analysis of semantic patterns in heterogeneous information networks for knowledge discovery across multiple domains. In our study, we consider an ontology as a domain and information retrieval across multiple ontologies in highly specialized medical domains as cross domain knowledge discovery. Any relationships across multiple domains (ontologies) are defined as cross domain relationships. Our model would be applicable to domains that have any common concepts, individuals or predicates (relationships) of ontologies. The building blocks that make up the best system of knowledge discovery with multiple domains are (i) a pattern based approach for predicate neighborhood defined for the heterogeneous information network, (ii) integrating the cross domain relations by evidences gathering from these patterns, (iii) graph partition and quantitative analysis using data mining algorithms, and (iv) exploration and discovery through query processing.

We demonstrate the cross domain knowledge discovery in the MedKDD framework using a case study with nine primary biological ontologies of Bio2RDF [17] including *ClinicalTrials* [18], *DrugBank* [19], *OMIM* [20], *PharmGKB* [21], *SIDER* [22], *KEGG* [23], *CTD* [24], *HGNC* [25], *MGI* [26]. We have implemented the MedKDD system and the experimental results clearly showed the validity of the MedKDD framework that was designed for Knowledge discovery from heterogeneous information networks across a medical domain.

The major content of this paper is organized as follows: We first present the MedKDD framework in Section Materials and Methods. We then describe the implementation of the MedKDD system and the experimental results in Section Results. We present discussion in Section Discussion. The conclusion and future work is discussed in Section Conclusion.

## Materials and Methods

We now present the MedKDD framework that aims to support knowledge discovery from cross domains by the construction of a hierarchy of topics in biomedical research. In the topic hierarchy, topics are analyzed for preserving neighboring information of relationships that are relevant in a given context (topic) in a heterogeneous information network. The topic models based on the predicates (relations) and their neighborhood patterns are defined as a graph in different levels of abstraction. We first rationalize a predicate-centric model *Cross Domain Neighborhood Patterns (CDNP)* that specifies high connectivity on the RDF/OWL graph for information sharing and integration. Second, we define the association measurement between predicates used in the CDNP patterns in the network. Third, we present the Predicate-based Hierarchical Agglomerative Clustering (PHAL) algorithm to cluster the heterogeneous information network based on the CDNP patterns.

### Cross Domain Neighborhood Patterns (CDNP)

In the MedKDD framework, the knowledge model is defined by levels of abstraction: (i) the smallest component is a predicate (relation) from a heterogeneous information network (RDF/OWL graphs), (ii) the intermediate component is a pattern that is defined by groups of predicates, (iii) at a higher abstraction level, a topic can be discovered from groups of patterns, and (iv) the highest level of abstraction that can be presented as an analytical view of multiple ontologies (cross domains). The relationships of domains can be determined from a comprehensive analysis of the discovered topics and patterns of predicates.

As the predicates define the relationships between subjects and objects, it is interesting to see that the relationships among subjects and objects are nicely defined through patterns and topics. In this paper, we define the Cross Domain Neighborhood Patterns (CDNP) that describe the association and collaboration among different predicates (relations) and concepts in heterogeneous information networks. In this analysis, only domain specific predicates are considered without considering OWL built-in predicates. There are two types of the CDNP patterns: *Cross-Domain Share* and *Cross-Domain Connectivity*.

**Definition 1: Cross-Domain Share Pattern** This pattern describes the resources sharing relationships between predicates where the resources are concepts from a heterogeneous information network (RDF graphs). Given two triples 〈*S*_*i*_, *P*_*i*_, *O*_*i*_〉, 〈*S*_*j*_, *P*_*j*_, *O*_*j*_〉, the conditions of the share pattern were defined as follows:

$$\begin{array}{ccc} & & \forall S_{i}\in D_{i},\;\forall P_{i}\in D_{i},\;\forall O_{i}\in D_{i}\;\text{and}\;\forall S_{j}\in D_{j},\;\forall P_{j}\in D_{j},\;\forall O_{j}\in D_{j}\\ & & \;\left(P_{i}\neq P_{j}\right)\&\&(S_{i}==S_{j}\left|\right|O_{i}==O_{j})\&\&\left(D_{i}\neq D_{j}\right). \end{array}$$

where the logical OR operator (||) returns the Boolean value true if either or both operands is true and returns false otherwise, the logical AND operator (&&) returns the Boolean value true if both operands are true and returns false otherwise. For all (denoted by ∀) *S*_*i*_, for all *P*_*i*_ and for all *O*_*i*_ are in a domain *D*_*i*_ and for all *S*_*j*_, for all *P*_*j*_, and for all *O*_*j*_ are in a domain *D*_*j*_, but these two domains *D*_*i*_ and *D*_*j*_ are different.

There are three types of Share patterns are defined as follows:

- The *Provider* pattern describes the relationship with a pair of predicates sharing a common object, describes the provider role of entity giving information to Consumers. This role has more out-degree edges than in-degree edges.
- The *Consumer* pattern describes the relationship with a pair of predicates sharing a common subject, describes the role of entity receiving information from Providers. Consumer has more in-degree edges than out-degree edges.
- The *Reacher* pattern describes the relationship with a pair of predicates having a same concept as a subject and object, describes the role connecting the Provider role with the Consumer role.

**Fig 1:**
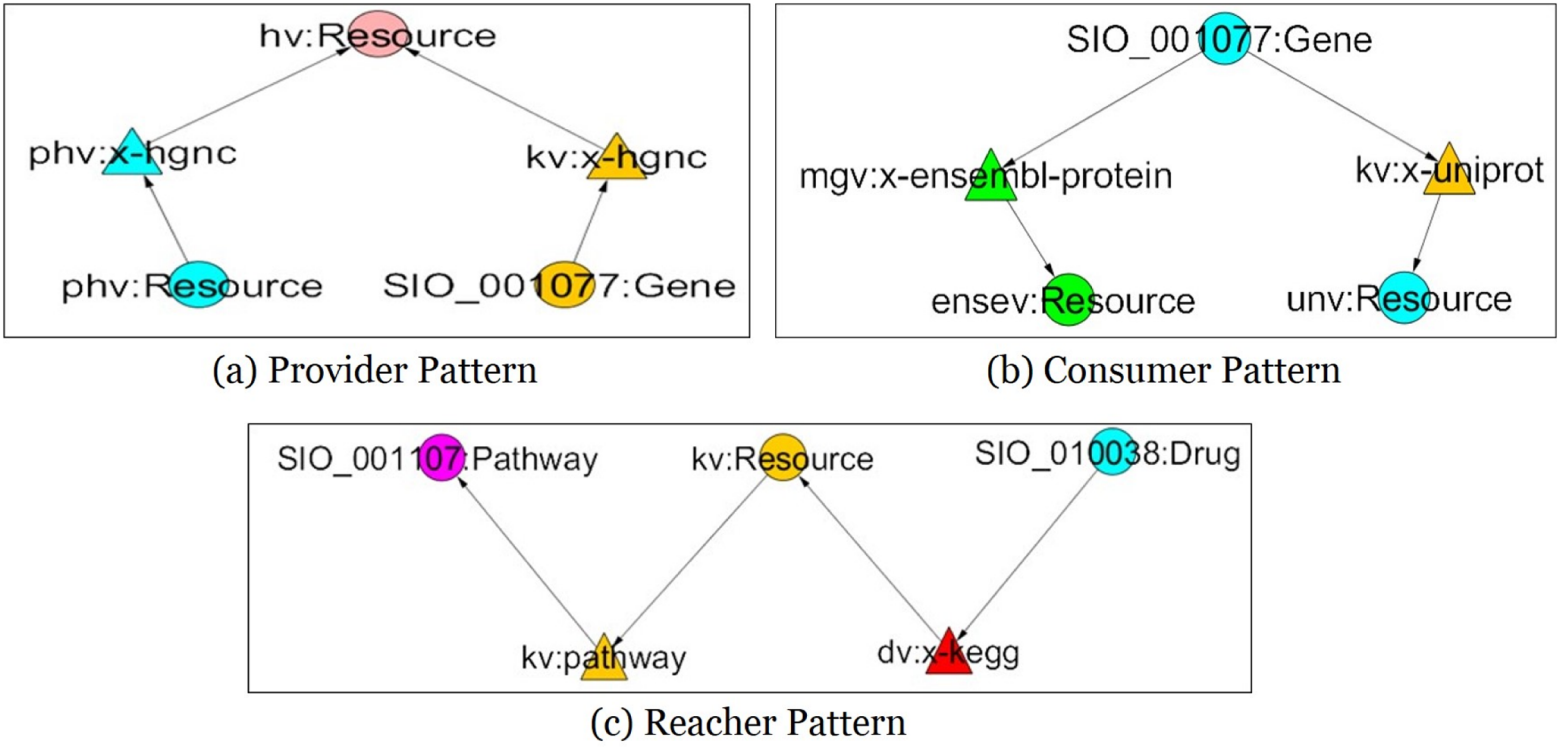
Cross Domain Share Patterns. Examples of three share patterns (a) *Provider*, (b) *Consumer*, (c) *Reacher* are shown in this figure. In this diagram, the circle represents a concept and the triangle represents a predicate.

Fig 1 shows the share patterns such that (a) Provider pattern: the object *hv:resource* is shared through two predicates *pv:x-hgnc* and *kv:x-hgnc* (b) Consumer pattern: the subject *SIO_001077:Gene* is shared with two predicates *mgv:x-ensembl-protein* and *kv:x-uniprot* (c) Reacher pattern: a concept *kv:Resource* is shared by two predicates *dv:x-kegg* and *kv:pathway*.

**Definition 2: Cross-Domain Connectivity Pattern** This pattern describes the connectivity relationships at least three predicates in a heterogeneous information network from different domains. This Connectivity pattern is defined using the *Reacher* pattern from Definition 1. A subject (*S*_*i*_) in a source domain (*D*_*i*_) is connected to an object (*O*_*i*_) in a target domain (*D*_*j*_) through cross-domain connectivity predicates (*P*_*i*_, *P*_*j*_ ∈ *P*_*c*_ and *D*_*i*_ ≠ *D*_*j*_). The pattern of the source domain or the target domain is defined as a *Reacher* pattern. There are two types of the Connectivity pattern: *Directional Connector* (DC) and *Non-Directional Connector* (NDC).

- The *DC* pattern describes the connectivity pattern considering the direction of the edges between predicates whose distance is higher than equal to 2.
- The *NDC* pattern is same with the DC pattern in terms of the predicate collaboration for indirect connectivity, however, the edge directions are not considered in this NDC pattern.

This Connectivity pattern is formally defined as follows: Given a *Reacher* pattern 〈*S*_*s*_, *P*_*s*_, *O*_*s*_〉 and a new triple 〈*S*_*i*_, *P*_*i*_, *O*_*i*_〉, the conditions of the connectivity pattern were as follows:

$$\begin{array}{ccc} & & \forall S_{s}\in D_{s},\;\forall P_{s}\in D_{s},\;\forall O_{s}\in D_{s}\;\text{and}\;\forall S_{i}\in D_{i},\;\forall P_{i}\in D_{i},\;\forall O_{i}\in D_{i}\\ & & \;\left(P_{s}\neq P_{i}\right)\&\&\left(O_{s}==S_{i}\right)\&\&\left(D_{s}\neq D_{i}\right). \end{array}$$

where the logical AND operator (&&) returns the Boolean value true if both operands are true and returns false otherwise. For all (denoted by ∀) *S*_*s*_, for all *P*_*s*_ and for all *O*_*s*_ are in a domain *D*_*s*_ and for all *S*_*i*_, for all *P*_*i*_, and for all *O*_*i*_ are in a domain *D*_*i*_, but these two domains *D*_*s*_ and *D*_*i*_ are different.

**Fig 2:**
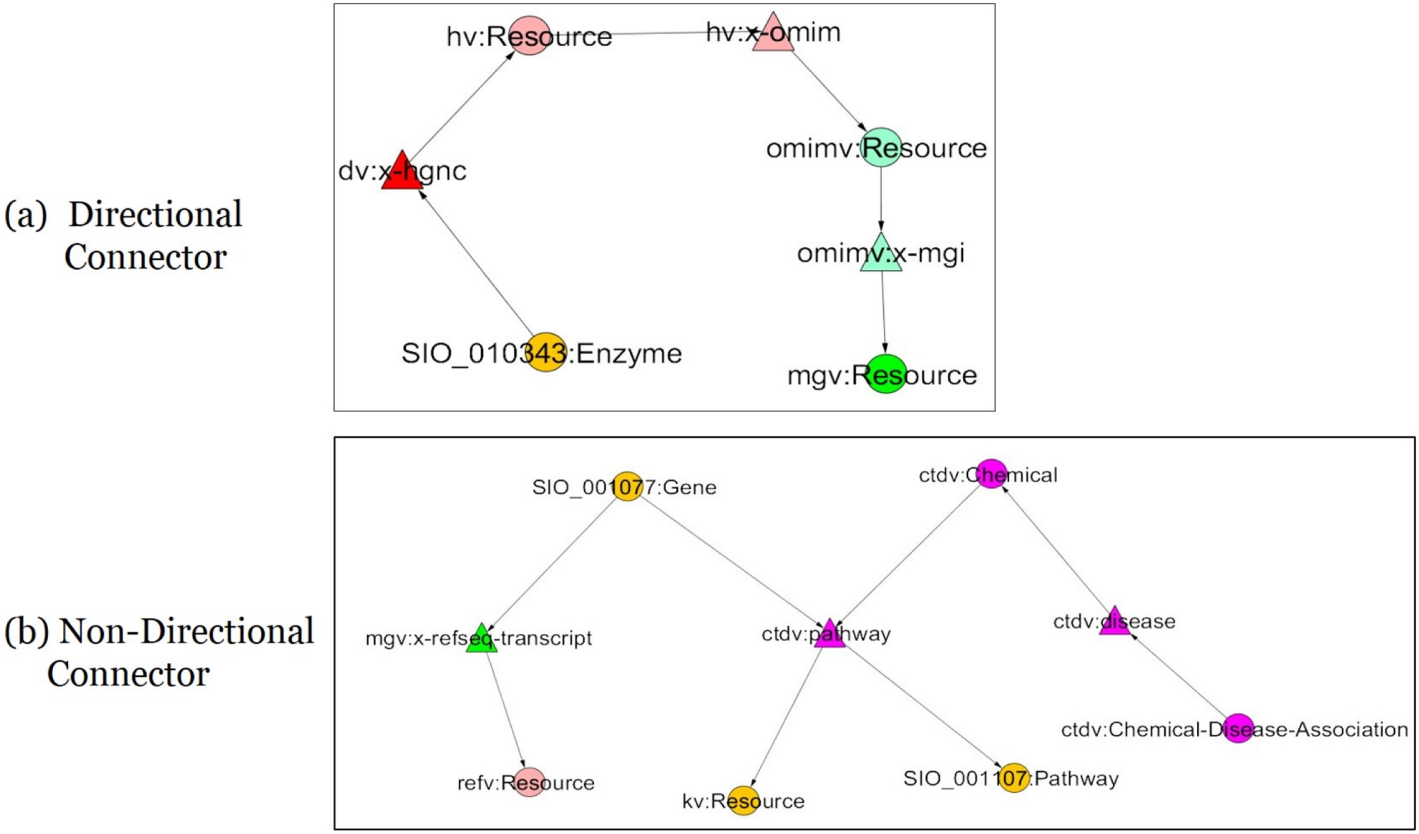
Cross Domain Connectivity Patterns. Two connectivity patterns (a) *Directional Connector* (DC) and (b) *Non-Directional Connector* (NDC) are shown in this figure. In this diagram, the circle represents a concept and the triangle represents a predicate. A color is assigned to each dataset as follows: DrugBank: Red; HGNC: Pink; MGI: Green; PharmGKB: Cyan; ClinicalTrials: Yellow; OMIM: Sky Blue; SIDER: Gray; KEGG: Orange; CTD: Magenta. The prefixes describe the domain of the concepts and predicates. ctdv:http://bio2rdf.org.ctd_vocabulary dv: http://bio2rdf.org/drugbank_vocabulary ensev: http://bio2rdf.org/ensembl_vocabulary hv: http://bio2rdf.org/hgnc_vocabulary kv: http://bio2rdf.org/kegg_vocabulary mgv: http://bio2rdf.org/mgi_vocabulary phv: http://bio2rdf.org/pharmgkb_vocabulary refv: http://bio2rdf.org/refseq_vocabulary unv: http://bio2rdf.org/uniprot_vocabulary.

Fig 2 shows the Connectivity patterns such that the subject and object are connected through three predicates: (a) Directional Connector (DC) among three predicates *dv:x-hgnc*, *hv:x-omim*, *ommimv:x-mgi* (b) Non-Directional Connector (NDC) among three predicates *mgv:x-refseq-transcript*, *ctdv:pathway*, and ctdv:disease.

**Definition 3: Topic** The *topic* describes bounded contexts through association patterns of both shared and connected predicates in a heterogeneous information network. Different topics may have completely different associations among any common predicates or concepts in heterogeneous domains. In a graph to represent the topic (called the topic graph), a group of predicates collaborate each other to share and connect information through the predicates of the CDNP patterns.

**Definition 4: Topic Boundary** The *topic boundary* (denoted as *B*) defines the scope of context in which the information can be associated and shared, and connected in a heterogeneous information network. The association and collaboration of information is described in terms of sets of concepts and relations within the given boundary on the heterogeneous information network.

Boundaries between contexts (topics) can be determined by various factors. Usually the dominant one is strongly associated with others so that this can be measured by high in-degree/out-degree and distance in a heterogeneous information network. This boundary can be set differently depending on the domains of interest. Multiple contexts can be found within the same domain context and similarly a single context can be founded across multiple domains. This paper focuses on the second kind of association.

The cross domain patterns are discovered with the bounded contexts which are a central concept in the knowledge discovery. The clustering technique is applied to partition a large and complex network into multiple smaller topics in the same context in an optimal manner. The bounded contexts are specifically tailored for a set of cross domain patterns. The boundary *B* is determined based on the distance *L* (without considering direction) between any two predicates.

**Definition 5: Degree of Diversity** The *degree of diversity* is defined to measure the degree of the association between predicates from different ontologies (domains) in a heterogeneous information network. The diversity degree is defined with an optimal weight assigned to links between predicates from different domains.

The weight will be computed to measure the degree of the association between predicates from different domains using the formula in Definition 6. The rationale is to capture diverse relations between predicates from multiple domains by giving a higher weight to the links across domains while giving a lower weight to links in a single domain.

**Definition 6: Cross Domain Diversity Weight** The weight represents the cross domain connectivity linking between predicates from different domains. This weight is computed based on the neighborhood predicates that are cross domains. For a given topic *T*_*i*_ with an average similarity association score $\bar{W_{i}}$, if a predicate pair {*p*_*i*_, *p*_*j*_} forms a cross domain relationship, i.e., *p*_*i*_ ∈ *D*_*i*_ *p*_*j*_ ∈ *D*_*j*_; *D*_*i*_ ≠ *D*_*j*_, *p*_*i*_, *p*_*j*_ ∈ *P* with an association score *w*_*ij*_, we define $w_{ij}^{\prime }$ as a cross domain association weight between predicates *p*_*i*_ and *p*_*j*_, such that

Let *DW*(*p*_*i*_, *p*_*j*_) be the diversity weight between two cross domain predicates *p*_*i*_, *p*_*j*_. Let *SW*(*p*_*i*_, *p*_*j*_) be the similarity weight between two predicates *p*_*i*_, *p*_*j*_ (without considering cross domain) Let $\bar{W_{p_{i}}}$ be the neighborhood association weight for a given predicate *p*_*i*_ (an average association weight with its neighborhood)

$$\begin{array}{c} DW\left(p_{i},p_{j}\right)=\{\begin{array}{cc} \max\left(\frac{SW\left(p_{i},p_{j}\right)+\bar{W_{p_{i}}}}{2},\frac{SW\left(p_{i},p_{j}\right)+\bar{W_{pj}}}{2}\right) & SW\left(p_{i},p_{j}\right)<\frac{SW\left(p_{i},p_{j}\right)+\bar{W_{p_{i}}}}{2}\\ SW(p_{i},p_{j}) & SW\left(p_{i},p_{j}\right)⩾\frac{SW\left(p_{i},p_{j}\right)+\bar{W_{p_{i}}}}{2} \end{array} \end{array}$$ (1)

**Fig 3:**
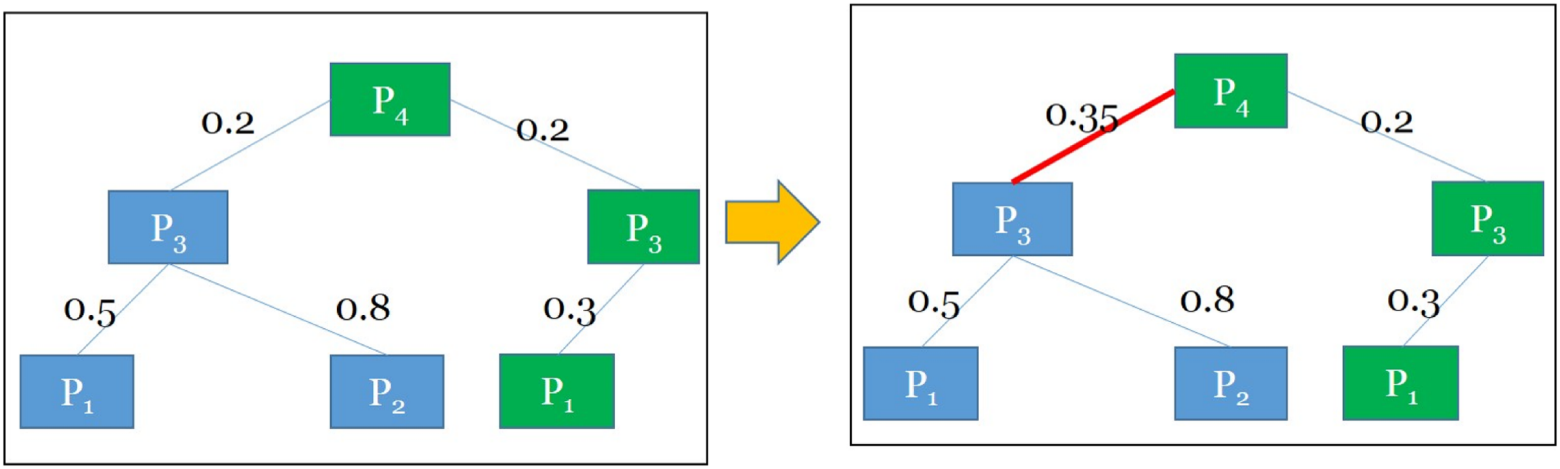
Cross Domain Diversity Weighting (Before/After). The cross domain diversity weight (DW) for the edge between predicate *P*_3_ and predicate *P*_4_ is computed as 0.35 using Eq (1). SW(*P*_3_, *P*_4_) = 0.2 and $\bar{W_{p_{3}}}$ = $\frac{0.2+0.5+0.8}{3}$ = 0.5 and $\bar{W_{p_{3}}}$ = $\frac{0.2+0.2+0.3}{3}$ = 0.23. DW(*P*_3_, *P*_4_) = $Max\left(\frac{SW\left(P_{3},P_{4}\right)+\bar{W_{p_{3}}}}{2},\frac{SW\left(P_{3},P_{4}\right)+\bar{W_{p_{4}}}}{2}\right)$ = $Max\left(\frac{0.2+\frac{0.2+0.5+0.8}{3}}{2},\frac{0.2+\frac{0.2+0.2+0.3}{3}}{2}\right)$ = *Max*(0.35,0.21) = 0.35.

In this paper, a threshold heuristic is employed to compute a topic boundary *B* for given datasets. We are encouraged by results on determining a topic boundary, where a heuristic has been devised increasing diverse association within a single topic on the topic boundary *B* as 3. The maximum distance between predicates (without considering the direction) in a topic is 3. For the given topic boundary *B* = 3, as shown in Fig 3, the cross domain diversity weight was computed for predicates *P*_3_ and *P*_4_ using Eq (1).

In this paper, we now present the relationships between domains that have been discovered by modeling the predicate neighborhood pattern and conducting the pattern-based topic discovery. Our work is related to the Ontology Alignment defined in [27] as a set of correspondences between two or more ontologies, corresponding relation holding according to a particular matching algorithm with classes, individuals, properties of ontologies.

**Definition 7: Domain Association** The *Domain Association* defines the association among domains that depicts a high level of views on cross domain collaboration. Based on the predicate collaboration in the CDNP patterns, the domain association and collaboration model can be defined. For each pattern, the top *K* predicates are considered to build the domain association model that represents the abstract relationships between these topics.

To describe the relationships between domains, three additional roles such as Bridger, Balancer, and Hub are defined.

- The *Bridger* role describes a collaborative relationship among domains in multiple domains and passes along information between them. This role plays a very important role to link two or more domains.
- The *Hub* role describes about a center of the domain, called the *influential* domains, that are strongly connected to other domains.
- The *Balancer* role describes the balanced collaboration in terms of receiving and producing information. The pattern can be identified based on the similar in-degree and out-degree edges of domain graphs.

### CDNP Association Measurements

We now define the measurement for the Cross Domain Neighborhood Patterns (CDNP) in terms of sets of concepts and relations (predicates) across the multiple domains. For this purpose, we describe how to quantify associations between different predicates across domains. It is based on the CDNP pattern describing the relationships between predicates *P*_*i*_ and *P*_*j*_ through a concept *C* across domains. The association measurement for the CDNP patterns varies based on different neighboring levels for each pair of predicates. Basically, we give a higher shared score to predicates with more shared concepts and lower scores to predicates with less shared concepts. Similarly, we give a higher connection score to closer predicates and lower scores to further predicates. We formally define the association measurement between predicates for the Cross-Domain Share patterns and Cross-Domain Connectivity patterns.

**Definition 8: Association Distance** The *association distance* defines the distance between associated predicates in a heterogeneous information network. Given a directed graph *G*(*C*, *P*), concepts *C* denote subject *S* and object *O* and *P* predicate in a RDF schema graph, respectively. Let *d*(*P*_*i*_, *P*_*j*_) represent the number of concepts *C* between *P*_*i*_ and *P*_*j*_. *r*(*P*_*i*_, *P*_*j*_) determines if a predicate *P*_*i*_ is reachable from another predicate *P*_*j*_ where the domain *D*_*i*_ of *P*_*i*_ is not the same from the domain *D*_*j*_ of *P*_*j*_, i.e., *D*_*i*_ ≠ *D*_*j*_, without considering the direction of links). *l*(*P*_*i*_, *P*_*j*_) indicates the shortest distance between *P*_*i*_ and *P*_*j*_.

$$\begin{array}{c} l\left(P_{i},P_{j}\right)=\{\begin{array}{cc} 0 & P_{i}=P_{j}\\ 1 & d(P_{i},P_{j})=1\\ L_{1}+L_{2} & L_{1}=d\left(P_{i},P_{k}\right),L_{2}=d\left(P_{k},P_{j}\right)\\ & r\left(P_{i},P_{k}\right)=true,r\left(P_{k},P_{j}\right)=true,r\left(P_{i},P_{j}\right)=true \end{array} \end{array}$$ (2)

The direct association describes the direct relationship between *P*_*i*_ and *P*_*j*_ in the distance *L* = 1 (without considering a direction) that is within the boundary *B*. The indirect association describes any relationship between *P*_*i*_ and *P*_*j*_ in distance *L* computed by Eq (2) within the boundary *B*, i.e., 1 < *L* ≤ *B*. The share pattern is the directed association while the Connectivity pattern is the indirect association. We now define these two probability based similarity scores: i) [*SA*](*P*_*i*_, *P*_*j*_) is defined a share pattern of any two predicates *P*_*i*_ and *P*_*j*_ ii) [*CA*](*P*_*i*_, *P*_*j*_) for a Connectivity pattern of any two predicates.

**Definition 9: Share Association** Given predicates *P*_*i*_ and *P*_*j*_ in a directed RDF schema graph *G*(*C*, *P*). Let *C*(*P*_*i*_) and *C*(*P*_*j*_) denote the entities (subjects or objects) that are directly connected to *P*_*i*_ and *P*_*j*_ regardless of the direction. *l*(*P*_*i*_, *P*_*j*_) is the reachability test for the given predicates *P*_*i*_, *P*_*j*_. *SA*(*P*_*i*_, *P*_*j*_) indicates the probability-based association matrix for a share pattern between *P*_*i*_ and *P*_*j*_.

$$\begin{array}{c} SA\left(P_{i},P_{j}\right)=\{\begin{array}{cc} 1 & l(P_{i},P_{j})=0\\ 0 & l\left(P_{i},P_{j}\right)\to \infty \left(nolink\right)\\ \frac{{\left(|C\left(P_{i}\right)\cap C\left(P_{j}\right)|\right)}^{2}}{|C\left(P_{i}\right)|*|C\left(P_{j}\right)|} & otherwise \end{array} \end{array}$$ (3)

**Definition 10: Connectivity Association** For a Connectivity pattern of any two predicates *P*_*i*_ and *P*_*j*_, *CA*(*P*_*i*_, *P*_*j*_) defines the probability-based association for a Connectivity pattern between *P*_*i*_ and *P*_*j*_ based on the Share Pattern. For the given Share Associations *SA*(*P*_*i*_, *P*_*k*_) and *SA*(*P*_*k*_, *P*_*j*_) and the distance between the predicates *l*(*P*_*i*_, *P*_*j*_), the connectivity association can be computed as follows:

$$\begin{array}{c} CA\left(P_{i},P_{j}\right)=\{\begin{array}{cc} SA\left(P_{i},P_{k}\right).SA\left(P_{k},P_{j}\right) & l(P_{i},P_{j})=2\\ \max_{1\leq k<j}CA\left(P_{i},P_{k}\right).CA\left(P_{k},P_{j}\right) & l(P_{i},P_{j})>2 \end{array} \end{array}$$ (4)

The definition is influenced by the chain matrix multiplication problem (a kind of dynamic programming) of determining the optimal sequence for performing a series of operations. After we get the similarity score for all pairs of predicates, we use the formula in Eqs (3) and (4) to generate a predicate association matrix for clustering.

**Definition 11: Predicate Association Matrix** Given the total number of predicates *n* and the probability-based association score for cross domain share patterns *SA*(*P*_*i*_, *P*_*j*_) and Cross Domain Connectivity Patterns *CA*(*P*_*i*_, *P*_*j*_) between predicates *P*_*i*_ and *P*_*j*_, *PA*[*P*_*i*_, *P*_*j*_] indicates an association matrix for all pairs of predicates *P*_*i*_ and *P*_*j*_

$$\begin{array}{c} PA\left[P_{i},P_{j}\right]=\{\begin{array}{cc} CA(P_{i},P_{j}) & l(P_{i},P_{j})>=2\\ SA(P_{i},P_{j}) & Otherwise \end{array} \end{array}$$ (5)

### Predicate-based Hierarchical Agglomerative Clustering

**Fig 4:**
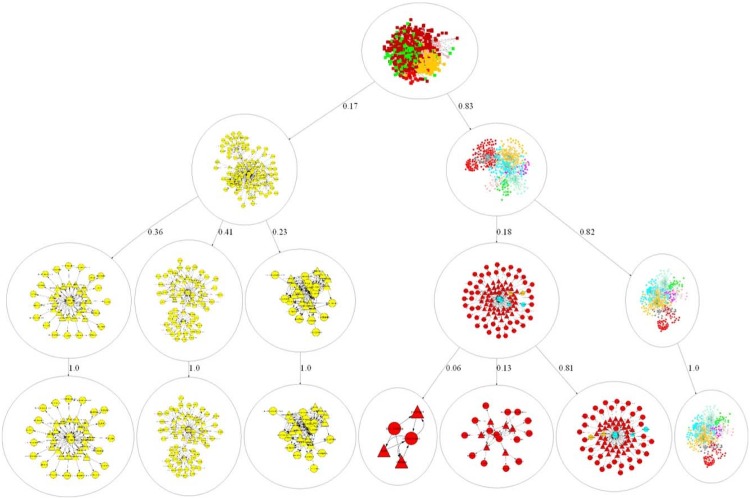
Top Down Topic Hierarchy Constructed by the Hierarchical Predicate-based K-Means clustering Algorithm. The top down topic hierarchy with three levels has seven topics at the third level. The number assigned to the edges indicates the distribution of predicates to its child node. The sum of the numbers should be one (e.g., 0.17 + 0.83 at the top level). A color is assigned to each domain as follows: DrugBank: Red; HGNC: Pink; MGI: Green; PharmGKB: Cyan; ClinicalTrials: Yellow; OMIM: Sky Blue; SIDER: Gray; KEGG: Orange; CTD: Magenta.

There are various different approaches in clustering heterogeneous information networks. In [28], we designed the Hierarchical Predicate-based K-Means clustering (HPKM) algorithm for discovery of relevant topics from integrated multiple sources and forms a topic hierarchy. The HPKM algorithm is an excellent way to summarize an integrated view of multiple ontologies as shown in Fig 4. However, we observe that HPKM is not suitable for cross domain knowledge discovery from heterogeneous information network. The reason is that the HPKM’s top-down approach focuses on global clustering based on homogeneous perspectives, however, ignoring the diverse and local perspectives of the network.

In this paper, we designed a new algorithm, called the Predicate-based Hierarchical Agglomerative Clustering (PHAL), for topic discovery from the heterogeneous information network of the multiple domains. PHAL is a hierarchical bottom-up clustering algorithm by applying Hierarchical Agglomerative clustering (HAC) [29] to the heterogeneous information network of cross domain ontologies. PHAL is creating a topic hierarchy through the analysis of the patterns quantified by the CDNP association measurement. PHAL starts with each predicate as a singleton cluster and then successively merges pairs of clusters while traversing up through its ancestors in the hierarchy.

**Fig 5:**
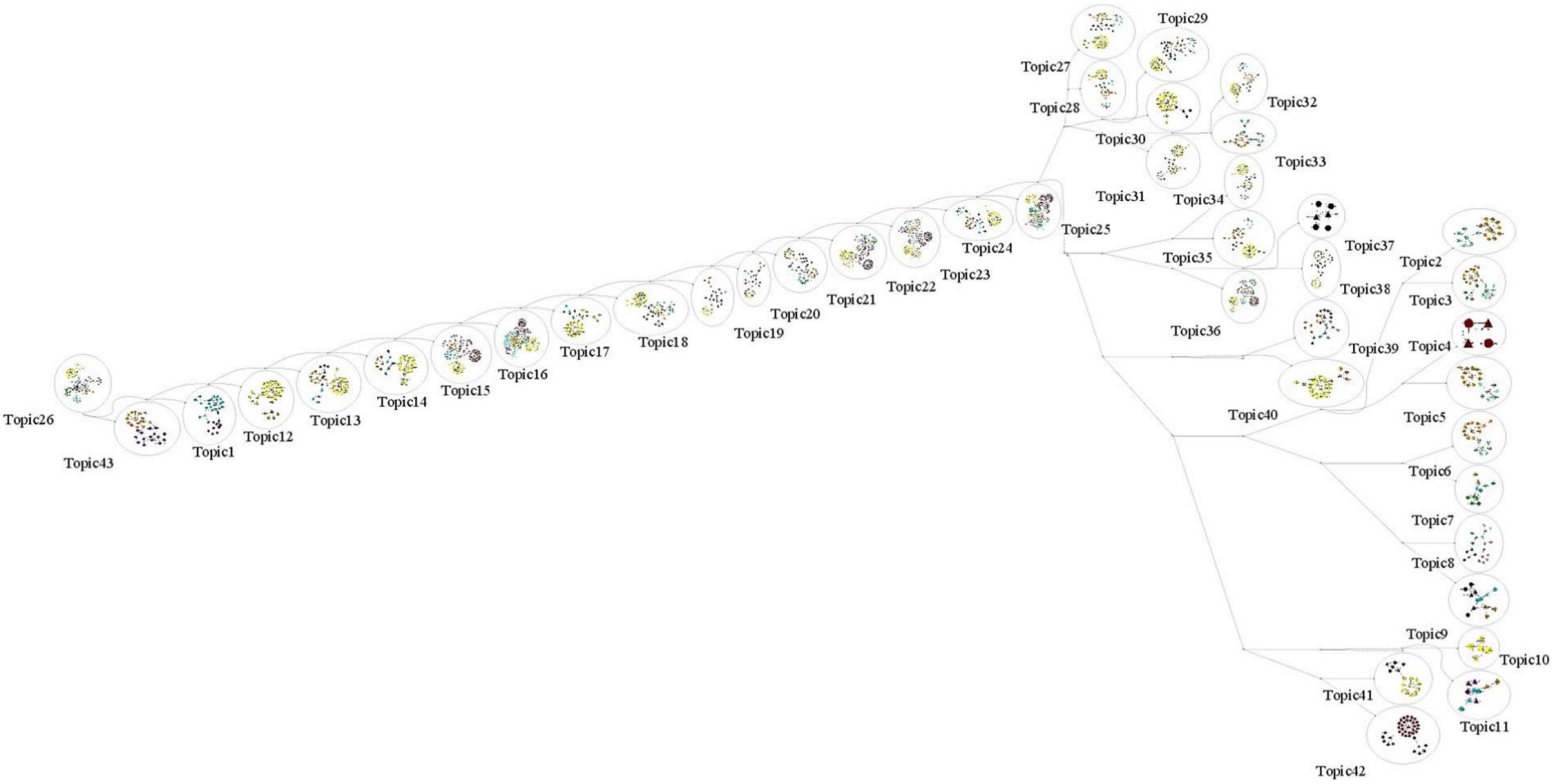
Bottom Up Topic Hierarchy Constructed by the Predicate-based Hierarchical Agglomerative Clustering Algorithm. Bottom up topic hierarchy with 43 topics. Topic ID is assigned to each cluster in this hierarchy. A color is assigned to each domain as follows: DrugBank: Red; HGNC: Pink; MGI: Green; PharmGKB: Cyan; ClinicalTrials: Yellow; OMIM: Sky Blue; SIDER: Gray; KEGG: Orange; CTD: Magenta.

Fig 5 shows a topic hierarchy generated from the PHAL algorithm. The PHAL algorithm has four phases as shown below and the pseudo codes are shown in Algorithms 1 and 2.

**Phase 1: Hierarchical Agglomerative Clustering** This phase focuses on clustering predicates from the heterogeneous information network of the given datasets using Hierarchical Agglomerative Clustering [29]. This algorithm is a bottom-up approach to build a hierarchy of topics based on the CDNP patterns until all predicates in the network belong to a topic group. The results from this learning process are a set of *topics* (*InitialMap*) in a hierarchical structure similar to the topics shown in Fig 5.

**Phase 2: Construction of Topics Starting at Level Mid** Given the tree from Phase 1, we first compute the mid-level of the tree (i.e., *Mid* = *H*/2, where *H* is the height of the hierarchy generated from Phase 1). The topics at the mid-level *Mid* are assigned to *FinalTopicSet*. If there is no topic at the level *Mid*, then go upward until find any topic groups on the subsequent level of the *Mid* (i.e., *Mid*-1) in the hierarchy. Among 43 topics shown in Fig 5, Topics 2-11 are the topic groups captured at the level *Mid*.

This phase illustrates the constructing process of topics for the remaining topics, which do not belong to the topic groups *InitialMap*. Starting from the level *Mid*—1, we start traversing the tree upward to construct topic groups with each topic at the the subsequent level of the *Mid* level (i.e., *Mid*—1) and assign it to *FinalTopicSet*. Repeat this step at *Mid*—2 until reaching the tree root. In addition, we have made a special topic group (i.e., *Topic*_1_) that is a collection of the singleton topics whose size is 1. Topics 12-43 in Fig 5 are newly constructed during this phase.

**Phase 3: Hierarchical Topic Refinement** There are some cases such that relevant concepts are disconnected. This is due to the hard partition in which a predicate was not allowed to join more than one topic. To handle the issue, a refinement process is conducted to construct a more complete topic model with the respective predicates and their neighborhood. More precisely, for any two pairs of predicates, if they form a Connectivity pattern and then we include their intermediate predicates to the topic and update those topics in *FinalTopicSet*. From this refinement process, a predicate may join more than one topic group that results into fuzzy clustering.

**Algorithm 1** Hierarchical Heterogeneous Clustering

**Input**: *X* = {*x*_1_, …*x*_*n*_}

**Output**: Topic Set *T* = {*t*_1_, …, *t*_*k*_}

/* **Phase 1: Hierarchical agglomerative clustering**

Define level *L* = 0

 Consider each element in *X* as a topic, save them in InitialMap with level *L* = 0

 Put pair 〈*L*, *X*〉 to InitialMap

 **While** *true* **do**

  **if** *The active set InitialMap only has one item* **then**

   break

  **else**

   Extract all topics at current level *L* from InitialMap

   Choose pair *p* and *q* ∈ *X* with the best distance computed using formula $\frac{1}{\left|p|*|q\right|}\sum_{m\in p}\sum_{n\in q}d\left(m,n\right)$

   *M* = *M* ∪ {*p*, *q*} //Save all pairs to set *M*

   *L* = *L* + 1 //Update the level

   **for** *each pair of elements p and q in set M* **do**

    Merge *p* and *q* into a new topic *u*

     Add *u* to set *X*

     Update InitialMap with 〈*L*, *X*〉

   **end**

  **end**

 **end**

/* **Phase 2: Construction of Topics Starting at Mid Level**

Get the tree height *L* determined from Phase 1

 Compute the middle level of the tree *Mid* = Roundup (*L*/2)

 //Construct the topics while traversing the tree upward until it reaches the tree root

 **while** Mid > 0 **do**

  **if** *There is at least one topic at level Mid of InitialMap* **then**

   Extract topics *T* = {*T*_1_, …, *T*_*i*_} at level *Mid* by checking 〈*Mid*, *T*〉 from InitialMap

   Define set *Z* containing all the remaining topics

   //Initializing the topic index

   index = |*T*| + 2 // Excluding *T*_1_ and the initial topics *T* = {*T*_1_, …, *T*_*i*_}

   FinalTopicSet = FinalTopicSet + *T*

   **for** *each topic* *z*_*i*_ in *Z* **do**

    **if** *z*_*i*_.*size* = 1 **then**

     Add *z*_*i*_ to the special topic *Topic*_1_

      Update FinalTopicSet with the special topic *Topic*_1_

    **else**

     Add *z*_*i*_ to *Topic*_*index*_

      Update FinalTopicSet with *Topic*_*index*_

      index++

    **end**

   **end**

   break

  **else**

   *Mid* = *Mid*-1

  **end**

**end**

return FinalTopicSet

**Algorithm 2** Hierarchical Topic Refinement

**Input**: FinalTopicSet = {*t*_1_, …, *t*_*k*_}

**Output**: FinalTopicSet =$\left\{t_{1}^{\prime },\ldots ,t_{k}^{\prime }\right\}$ //refined topics with new predicates

/* **Phase 3: Hierarchical Topic Refinement**

**for** *each topic t in FinalTopicSet* **do**

 **for** *any two predicates p_i_ and p_j_ in topic t* **do**

  **if** *p*_*i*_ *and* *p*_*j*_ *are connected through a Connectivity pattern & d(p_i_, p_j_) = 2* **then**

   find the intermediate predicate *p*_*t*_ between *p*_*i*_ and *p*_*j*_

    add predicate *p*_*t*_ to topic *t*

   **end**

  **if** *p*_*i*_ *and* *p*_*j*_ *are connected through a Connectivity pattern & d(p_i_, p_j_) = 3* **then**

   find the two intermediate predicates *p*_*m*_ and *p*_*n*_ between *p*_*i*_ and *p*_*j*_

    add predicate *p*_*m*_ to topic *t*

    add predicate *p*_*n*_ to topic *t*

  **end**

 **end**

**end**

## Results

### Implementation

The MedKDD system was implemented using Java in Eclipse Juno Integrated Development Environment [30]. Apache Jena API [31] was used to analyze multiple ontologies in OWL. We used R computing environment [32] for our experimental validation and implemented a software plugin for query and schema graph visualization using CytoScape 3.0.2 [33]. In addition, we have built a SPARQL query endpoint on a single machine that is hosted at the UMKC Distributed Intelligent Computing (UDIC) lab. The OPEN LINK Virtuoso server version 6.1.3 was installed and the nine domains (*ClinicalTrials* [18], *DrugBank* [19], *OMIM* [20], *PharmGKB* [21], *SIDER* [22], *KEGG* [23], *CTD* [24], *HGNC* [25], *MGI* [26]) were imported into the graph domain http://Bio2RDF.com#. The endpoint for SPARQL query services is http://134.193.129.248:8890/isparql/.

**Fig 6:**
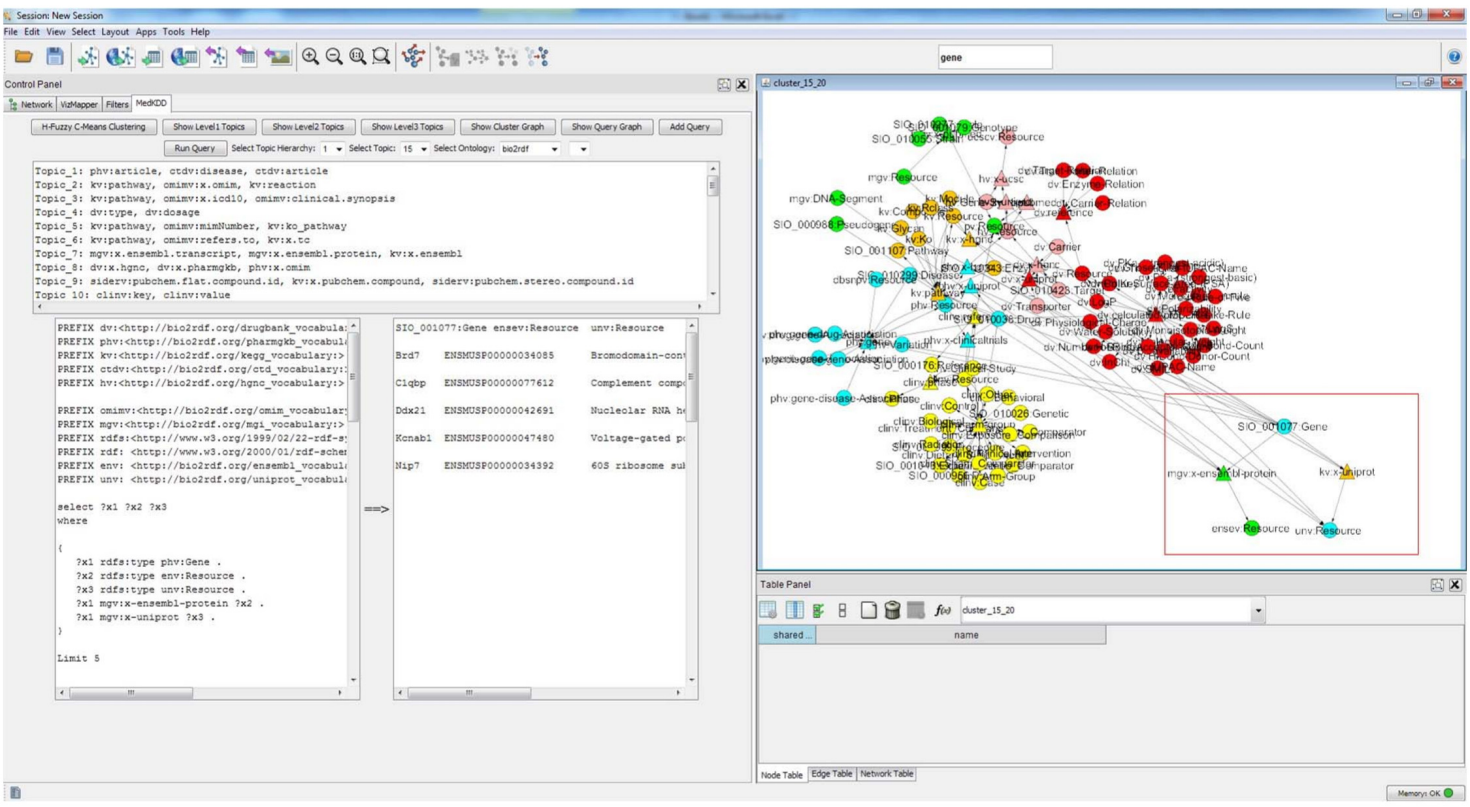
MedKDD Tool: Cross Domain Knowledge Discovery. The top left panel shows the list of topics in Bio2RDF Topic 15. The right panel shows the visualization of one of the topics discovered from this dataset. The bottom left panel show the SPARQL query for a selected topic and the query results from the execution of the selected query.

Fig 6 shows the MedKDD tool that are designed for browsing the generated topics and performing the interactive query design and processing. The tool shows the list of topics generated from the nine ontologies in OWL. For a selected topic, questions both in free text and SPARQL query format will be automatically generated. The topic graph and query graph can be visualized for the selected query. When the query button is clicked, the SPARQL query will be executed and the query output will be shown in the bottom right box. Then, the corresponding topic graph will be displayed on the canvas in the right panel. Moreover, by clicking the query graph button, the relevant concepts and predicates in the SPARQL query will also be highlighted as seen in Fig 6.

### Topic Discovery in Cross Domains

For the given nine ontologies in OWL shown in Table 1, we have conducted the pattern analysis for topic discovery. We have computed the rankings of predicates, patterns, and topics discovered from our knowledge discovery process and also summarized the relationships among domains based on the discovered patterns and topics.

**Fig 7:**
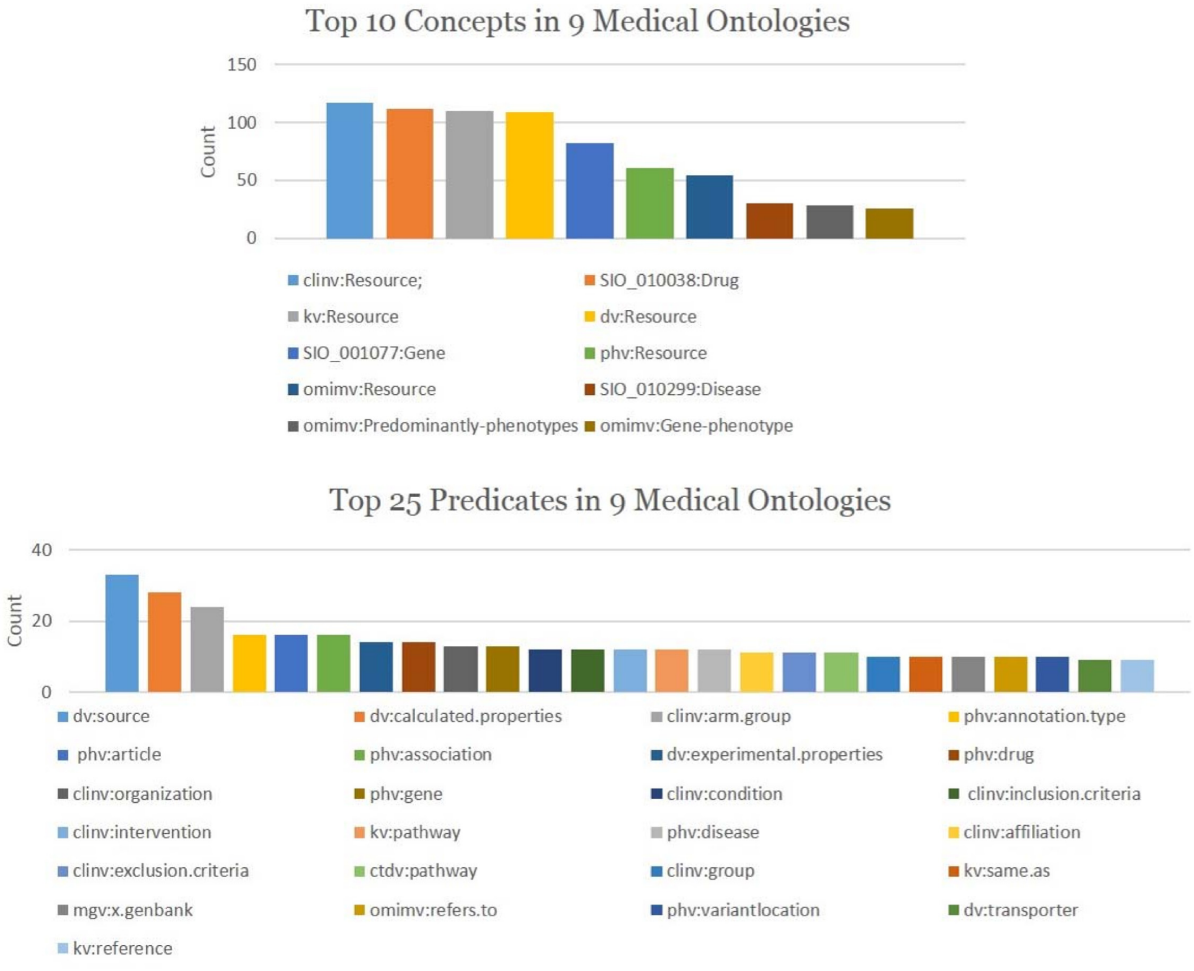
Top Concepts and Predicates. (a) Top 10 Concepts (b) Top 25 Predicates.

**Fig 8:**
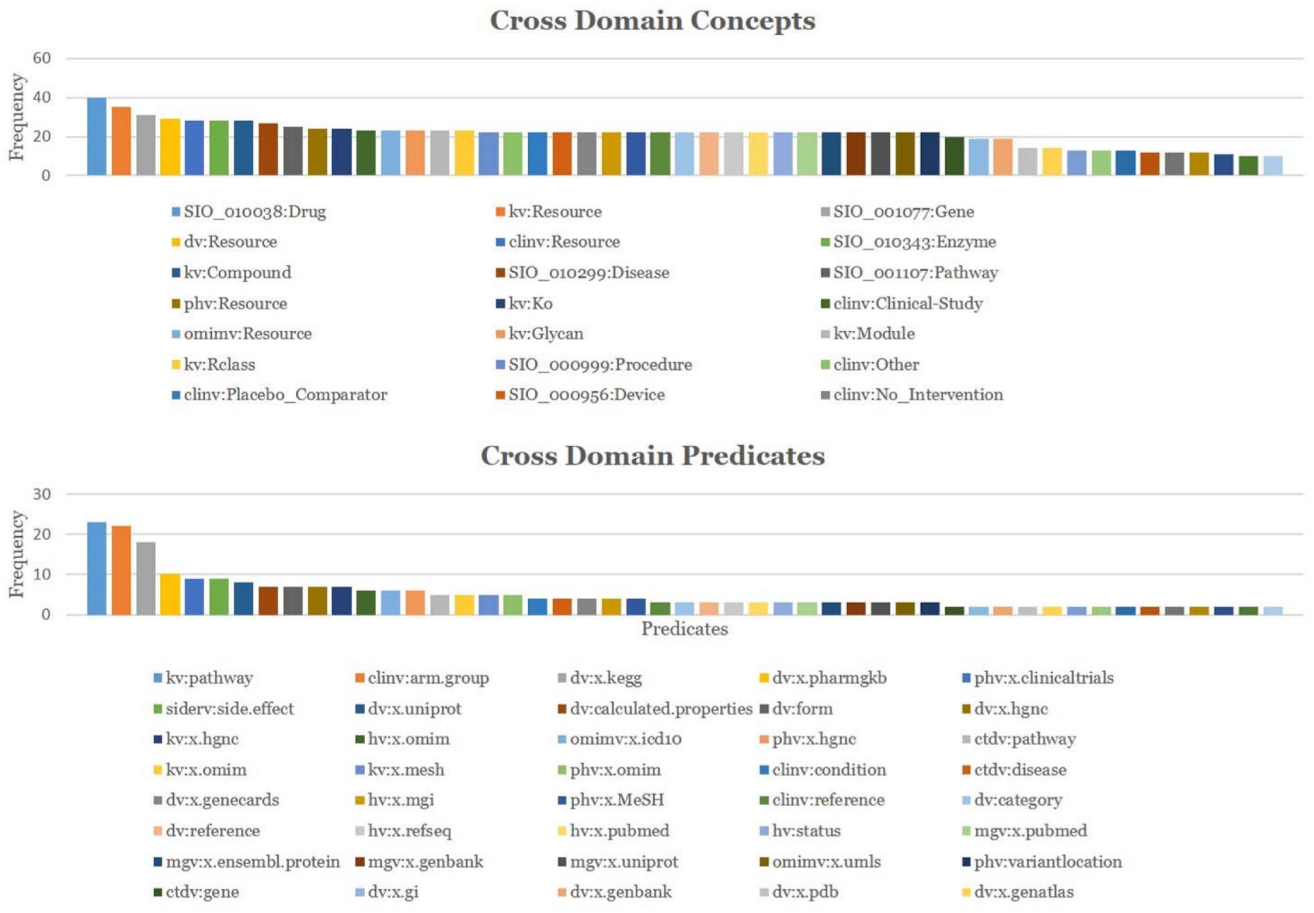
Cross Domain Topic Ranking. (a) Feature-based Ranking (b) Pattern-Based Ranking. Popularity is measured by In-degree/Out-degree of predicates. Verity is measured by the number of domains involved. The numbers in the bar graph are the topic ID (ranged: 1—43).

**Fig 9:**
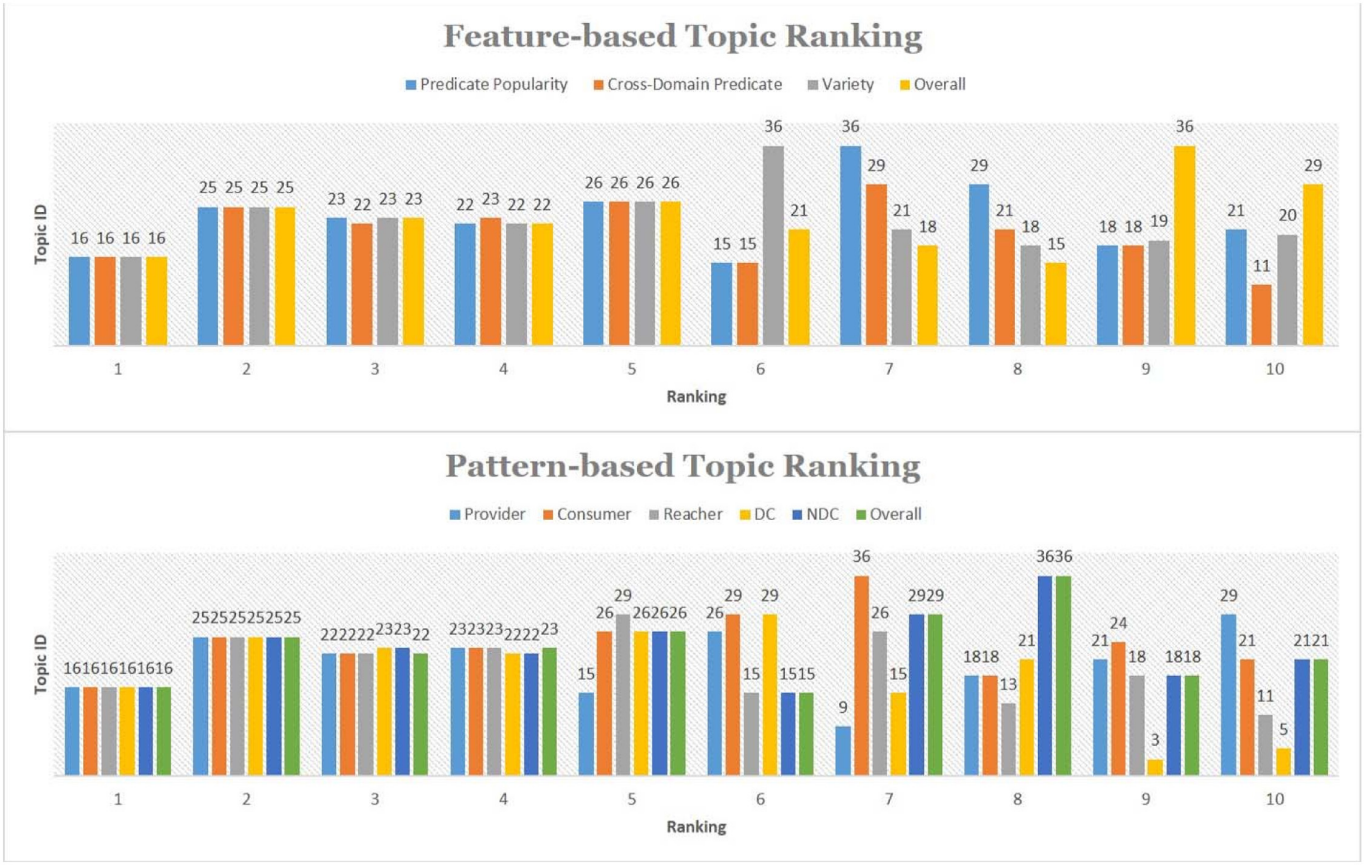
Cross Domain Concept and Predicate Ranking. (a) Top 40 Concepts (b) Top 40 Predicates. The prefixes describe the domain of the concepts and predicates. clinv: http://bio2rdf.org/clinicaltrials_vocabulary ctdv:http…bio2rdf.org.ctd_vocabulary dv: http://bio2rdf.org/drugbank_vocabulary hv: http://bio2rdf.org/hgnc_vocabulary kv: http://bio2rdf.org/kegg_vocabulary mgv: http://bio2rdf.org/mgi_vocabulary omimv: http://bio2rdf.org/omim_vocabulary phv: http://bio2rdf.org/pharmgkb_vocabulary sider: http://bio2rdf.org/sider_vocabulary.

- **CDNP Patterns in Topic Discovery** An analysis is conducted to gain a better understanding of the CDNP patterns in cross domain topic discovery. Table 2 shows the Cross Domain Neighborhood Patterns (CDNP) discovered from 43 topics: 1676 *Provider* Patterns, 5953 *Consumer* Patterns, 3572 *Reacher* Patterns, 1990 *Directional Connector* patterns and 14434 *Non-Directional Connector* patterns. Interestingly, 77% of the CDNP patterns we discovered are cross domain (50% of the *Provider* patterns, 45% of the *Consumer* Patterns, 40% of the *Reacher* Patterns, 100% of the *Directional Connector* patterns, and 100% of the *Non-Directional Connector* patterns). The share patterns in the lower level are part of the Connectivity patterns in the higher level. From these results, we confirm that the CDNP patterns play a significant role in integrating data and finding cross domain topics from heterogeneous information networks.
- **Predicate and Concept Ranking**: The predicates (the primary component in MedKDD) and their associated concepts are ranked based on their in-degree and out-degree. From this analysis, we found out the roles of ontologies for cross domain collaboration in heterogeneous information networks. Among 374 concepts, top concepts such as *clinv:Resource*, *kv:Resource*, *dv:Resource*, *phv:Resource* are shown in Fig 7(a). As shown in Fig 7(b), among 330 predicates, top 10 predicates such as *dv:source* and *dv:calculated.properties* are from three ontologies such as *DrugBank*, *ClinicalTrials*, and *PharmGKB*. These predicates and concepts are mainly from the primary ontologies including *ClinicalTrials*, *KEGG*, *DrugBank*, and *PharmGKB*.
- **Cross Domain Predicate and Concept Ranking**: The contents of cross domains were ranked based on the in-degree/out-degree of cross domain concepts and predicates. We observed the cross domain rankings with predicates and concepts were different from the non-cross domain rankings. However, the ontologies playing important roles are similar. Fig 8 shows 40 cross domain concepts and predicates. Among them, SIO:Drug, kv:Resource and SIO:Gene are top three cross domain concepts of *PharmGKB* (SIO normalized), *KEGG*, and *DrugBank* (SIO normalized). *kv:pathway*, *clinv:arm.group* and *dv:x.kegg* are top three cross domain predicates of *KEGG*, *ClinicalTrials*, and *DrugBank*, respectively.
- **Topic Ranking with Cross Domain Features**: These patterns are ranked according to primary features such as cross domain predicates, predicate popularity (in-degree/out-degree of the predicates), and domain verity (the number of domains in which the patterns are captured). Fig 9(a) shows top 5 topics (Topic 16, Topic 25, Topic 23, Topic 22 and Topic 26) computed by the cross domain features. Table 3 shows the top 3 predicates and top 2 unique predicates of these topics.
- **Topic Ranking with Cross Domain Neighborhood Patterns**: Topics are ranked based on the CDNP patterns. Fig 9(b) shows top five topics (Topic 16, Topic 25, Topic 23, Topic 22 and Topic 26). The ranking based on the counts of the CDNP patterns (*Provider*, *Consumer*, *Reacher*, *CD* and *NCD* patterns) is very similar to the ranking computed by the predicate popularity, cross domain predicate, and variety shown in Fig 9(a). This confirms that the proposed pattern-based approach reflects an excellent understanding of the important features of the network such as density, verity, and popularity.

**Table 1:** Case Study Datasets: Ontologies.

Framework	Dataset	P#	C#	T#	Description
MedKDD	ClinicalTrials (Yellow)	56	62	486	database of publicly and privately supported clinical studies of human participants conducted around the world. http://download.bio2rdf.org/release/3/clinicaltrials/clinicaltrials.html
MedKDD/SLAP	CTD (Magenta)	14	19	74	cross-species chemical-gene/protein interactions and chemical- and gene-disease relationships to illuminate molecular mechanisms underlying variable susceptibility and environmentally influenced diseases. http://download.bio2rdf.org/release/3/ctd/ctd.html
MedKDD/SLAP	DrugBank (Red)	63	92	401	bioinformatics and chemoinformatics resource that combines detailed drug (i.e. chemical, pharmacological and pharmaceutical) data with comprehensive drug target (i.e. sequence, structure, and pathway) information. http://download.bio2rdf.org/release/3/drugbank/drugbank.html
MedKDD/SLAP	HGNC (Pink)	14	16	34	unique and meaningful names to every human gene. http://download.bio2rdf.org/release/3/hgnc/hgnc.html
MedKDD/SLAP	KEGG (Orange)	72	61	299	an integrated database resource consisting of 16 main databases, broadly categorized into biological systems information, genomic information, and chemical information. http://download.bio2rdf.org/release/3/kegg/kegg.html
MedKDD	MGI (Green)	14	20	68	data on gene characterization, nomenclature, mapping, gene homologies, among mammals sequence links, phenotypes, allelic variants and mutants, and strain data. http://download.bio2rdf.org/release/3/mgi/mgi.html
MedKDD/SLAP	OMIM (Light Green)	35	30	175	a comprehensive, authoritative, and timely compendium of human genes and genetic phenotypes. The full-text, referenced overviews in OMIM contain information on mendelian disorders and over 12,000 genes. OMIM focuses on the relationship between phenotype and genotype. http://download.bio2rdf.org/release/3/omim/omim.html
MedKDD	PharmGKB (Cyan)	47	60	218	PharmGKB curates primary genotype and phenotype data, annotates gene variants and gene-drug-disease relationships via literature review, and summarizes important PGx genes and drug pathways. http://download.bio2rdf.org/release/3/pharmgkb/pharmgkb.html
MedKDD/SLAP	SIDER (Gray)	15	14	82	SIDER contains information on marketed medicines and their recorded adverse drug reactions. The information include side effect frequency, drug and side effect classifications and links to further information (e.g., drug-target relations). http://download.bio2rdf.org/release/3/sider/sider.html
	Total	330	374	1837	Cross domain data model based on these 9 datasets

In this table, each ontology is assigned with a color (for example, the color of CriticalTrials is yellow) that is used in a topic/patten graph. There are six common datasets (DrugBank, HGNC, SIDER, OMIM, KEGG, CTD) between MedKDD and SLAP. P: Predicates, C: Concepts, T: Triples. Some of the built-in OWL/RDF concepts and predicates are omitted in our research. The information in this table is extracted from the Bio2RDF project http://download.openbiocloud.org/release/3/release.html

**Table 2:** Cross Domain Neighborhood Patterns.

Patterns	Share Pattern			Connection Pattern		
	Provider	Consumer	Reacher	DC	NDC	Total
Total Patterns	1676	5953	3572	1990	14434	27625
Cross Domain Patterns	842 (50%)	2690 (45%)	1432 (40%)	1990 (100%)	14434 (100%)	21388 (77%)

Cross Domain Patterns per type of the CDNP patterns (Provider, Consumer, Reacher, Directional Connector and Non-Directional Connector)

**Table 3:** Top 5 Cross Domain Topics.

Topic#	Predicate#	Top 3 Predicates	Top 2 Unique Predicates
Topic 16	119	dv:source; dv:calculated.properties; clinv:arm.group	phv:drug; phv:disease
Topic 25	72	dv:calculated.properties; clinv:arm.group; phv:annotation.type	phv:association; phv:article
Topic 23	39	dv:calculated.properties; clinv:arm.group; kv:pathway	clinv:group; kv:module
Topic 22	36	dv:calculated.properties; clinv:arm.group; kv:pathway	pathway; dv:x.uniprot
Topic 26	24	clinv:arm.group; kv:pathway; mgv:x.genbank	dv:transporter; dv:target

For top five topics (Topic 16, Topic 25, Topic 23, Topic 22 and Topic 26), #predicates, top three predicates and top two unique predicates were specified. The top predicates were computed based on the in-degree/out-degree of these predicates.

### Comparative Analysis for Cross Domain Knowledge Discovery

The comparative analysis will provide valuable insight into the effectiveness of the Cross Domain Neighborhood Patterns (CDNP) and the CDNP-based topic discovery model. The evaluation of the proposed model has been conducted using practical examples of the cross domain predicate patterns and topic discovery. We show the patterns are useful in knowledge discovery from multiple ontologies through evaluation and validation of the proposed model compared to other approaches in knowledge discovery from diverse domains.

#### Comparative Analysis: Top Down Clustering vs. Bottom Up Clustering

The case studies involve the comparative analysis with the HPKM and PHAL algorithms and experiments with the both algorithms to confirm the effectiveness of the proposed method. For the given nine ontologies shown in Table 1, we have conducted the topic discovery by applying the proposed PHAL algorithm and the HPKM algorithm. As mentioned previously, HPKM is an excellent way to summarize an integrated cross-domain ontologies, as shown in Fig 4. However, HPKM could not capture interesting patterns from heterogeneous information networks of cross domains. From the HPKM analysis in Table 4, only seven coarse grained topics were discovered and two of them are cross domain. It is because predicates from a single domain are strongly related compared to ones from cross domain. From the PHAL analysis in Table 4, we found 43 topics from the heterogeneous information networks of the given cross domains and 93% of the discovered patterns (40 topics are cross domains and 3 topics are single domain) are cross domains. In addition, we computed the average predicate number per topic, the average in-degree and output-degree per topic, the average density per topic and the association score per topic. The density was computed using $D=\frac{2E}{N(N-1)}$ where *N* is the number of nodes (concepts and predicates) and *E* is the number of edges (links between nodes). The association score were computed by the Predicate Association formula Eq (5). Zero is defined as the smallest number. The closer to zero, the smaller it is. The results demonstrate the PHAL algorithm provides superior outcomes compared with HPKM in topic discovery from heterogeneous information networks.

**Table 4:** Cross Domain Clustering: PHAL vs. HPKM.

Features	PHAL	HPKM
Topic #	43	7
Cross Domain Topic #	40	2
Average Diversity (Domain#)	4.14	2.28
Total Predicate Size	539	330
Average Predicate Size per Topic	12.5	47.14
Average In-degree and Out-degree per Topic	45(I) 30(O)	142(I) 89(O)
Average Density per Topic	252	490
Average Predicate Association Score	0.42	0.70

Comparison between Top-down Clustering (HPKM—Hierarchical Predicate-based K-Means Clustering) and Bottom-up Clustering (PHAL—Predicate-based Hierarchical Agglomerative Clustering). In PHAL, the fuzzy clustering is allowed for predicates so that the predicates may appear in more than one topic. The density was computed using $D=\frac{2E}{N(N-1)}$ where *N* is the number of nodes (concepts and predicates) and *E* is the number of edges (links between nodes). The association score were computed by the Predicate Association formula Eq (5). Zero is defined as the smallest number. The closer to zero, the smaller it is.

Table 5 shows that there are 330 unique predicates and 275 unique concepts. Interestingly, about 88% of the predicates and 65% of the concepts are cross domain. Fig 7(a) and 7(b) show top 10 concepts and top 25 predicates, respectively. Fig 8(a) and 8(b) show the top 40 cross domain concepts and predicates, respectively. The nine ontologies used in our case study show high potentials to be used for cross-domain analysis and linking for semantic interoperability.

**Table 5:** Cross Domain Concepts and Predicates before/after Clustering.

Feature	Before Clustering			After Clustering	CDNP Pattern		Count per Topic		
	Unique	Total	Cross Domain	Total	Share	Connectivity	Average	Max	Min
Predicates	330	330	291	539	329	330	12.5	119	2
Concepts	275	374	243	1745	275	374	40.6	181	2

The predicate/concept count before and after clustering. Many of them are cross domain that can be easily associated with concepts/predicates from other domains. After the clustering, both concepts and predicates are duplicated (fuzzy clustering). The concepts/predicate counts for share and connectivity patterns are reported. In addition, average, min and max of concepts and predicates per topic are reported.

As seen in Table 5, about 26% of concepts (99 out of 374) appear in more than one domain even before the clustering while all 330 predicates are unique (this means each predicate appears in only one domain among 9 domains). Specifically, a generic concept like *Resource* appears 92 times and *pubmed_vocabulary:Resource* appears in all 9 domains. This indicates that concepts like *Resource* are mainly used for a high level mapping between different domains. Thus, these concepts are too abstract to be of practical use of such data. For the data integration, data normalization was performed to map 30 Semanticscience Integrated Ontology (SIO) concepts to domain concepts. In addition, about 45% (149 of 330 predicates) are named with a prefix *x*. This indicates that the predicates are also too abstract to provide meaningful relationships between concepts. After clustering, the size of predicates became doubled and the concepts quintupled. All the predicates except *sider_vocabulary:reported.frequency* are fully contributed to the integration of cross domains and discovery of relevant patterns. Through the normalization and clustering, relevant concepts and predicates were integrated and clustered according to their contexts.

#### Comparative Analysis: MedKDD vs. SLAP

We have conducted a comparative analysis with the Semantic Link Association Prediction (SLAP) [34] that was designed for detecting drug target association. This experiment was designed to compare between SLAP and MedKDD in terms of their capacity in handling cross topic and cross domain knowledge discovery using the six common datasets of MedKDD and SLAP shown in Table 1. MedKDD has an advanced capability on information retrieval for the relationships between two concepts, e.g., 〈Drug → Gene〉, the relationships among multiple concepts across topics, e.g., 〈Drug → Target → Gene〉, and the relationships across domains (DrugBank and OMIM), e.g., {DrugBank:〈Drug → Target〉 ⇒ *OMIM*:*Uniprot*}, where the symbol → represents a path from one concept to another within a single domain and the symbol ⇒ represents a path from one concept to anther across domain. Similarly, SLAP also has the ability to retrieve the information on the association between drugs and targets. However, SLAP does not support the information retrieval for any other association besides the drug and target association.

First, we have conducted several queries that are designed to retrieve the association among the key concepts in *DrugBank* (i.e., *drug*, *target*, *gene*). In order to demonstrate the knowledge discovery process with multiple datasets, the top five drug instances such as *NADH*, *Beta-D-Glucose*, *Flavin adenine dinucleotide*, *Pyridoxal Phosphate*, and *Citric Acid* shown in Table 6 were selected among 6071 possible drug instances in terms of the number of targets and their associated genes. As seen from Table 6, one drug may have *m* targets and then *m* targets relate to *n* genes, where m>n>0.

**Table 6:** Top 5 Drug Instances & Cross Topic Query Results of MedKDD & SLAP.

Drug Name	MedKDD		SLAP	
	#Targets	#Genes	#Targets	#Genes
NADH [drugbank:DB00157]	143	141	0	0
Beta-D-Glucose [drugbank:DB02379]	90	11	0	0
Flavin adenine dinucleotide [drugbank:DB03147]	80	15	0	3
Pyridoxal Phosphate [drugbank:DB00114]	66	54	0	56
Citric Acid [drugbank:DB04272]	64	12	0	0

This table shows the cross topic query performance for MedKDD and SLAP. #Targets indicates the results from the query like *dv:Drug (SIO_010038)* → *dv:target* → *dv:Target(SIO_010423)*. #Genes indicates the results from the query like *dv:Drug (SIO_010038)* → *dv:target* → *dv:Target(SIO_010423)* → *dv:x-genecards* → *dv:Gene(SIO_001121)*. The query about the drug and target association is from a single topic, Bio2RDF Topic 27. However, the query about the drug, target and gene association is a query across topics between Bio2RDF Topic 16 (Gene) and Bio2RDF Topic 27 (Drug and Target). MedKDD retrieved all the relevant information for both queries while SLAP retrieved partial information about drug and gene association.

**Fig 10:**
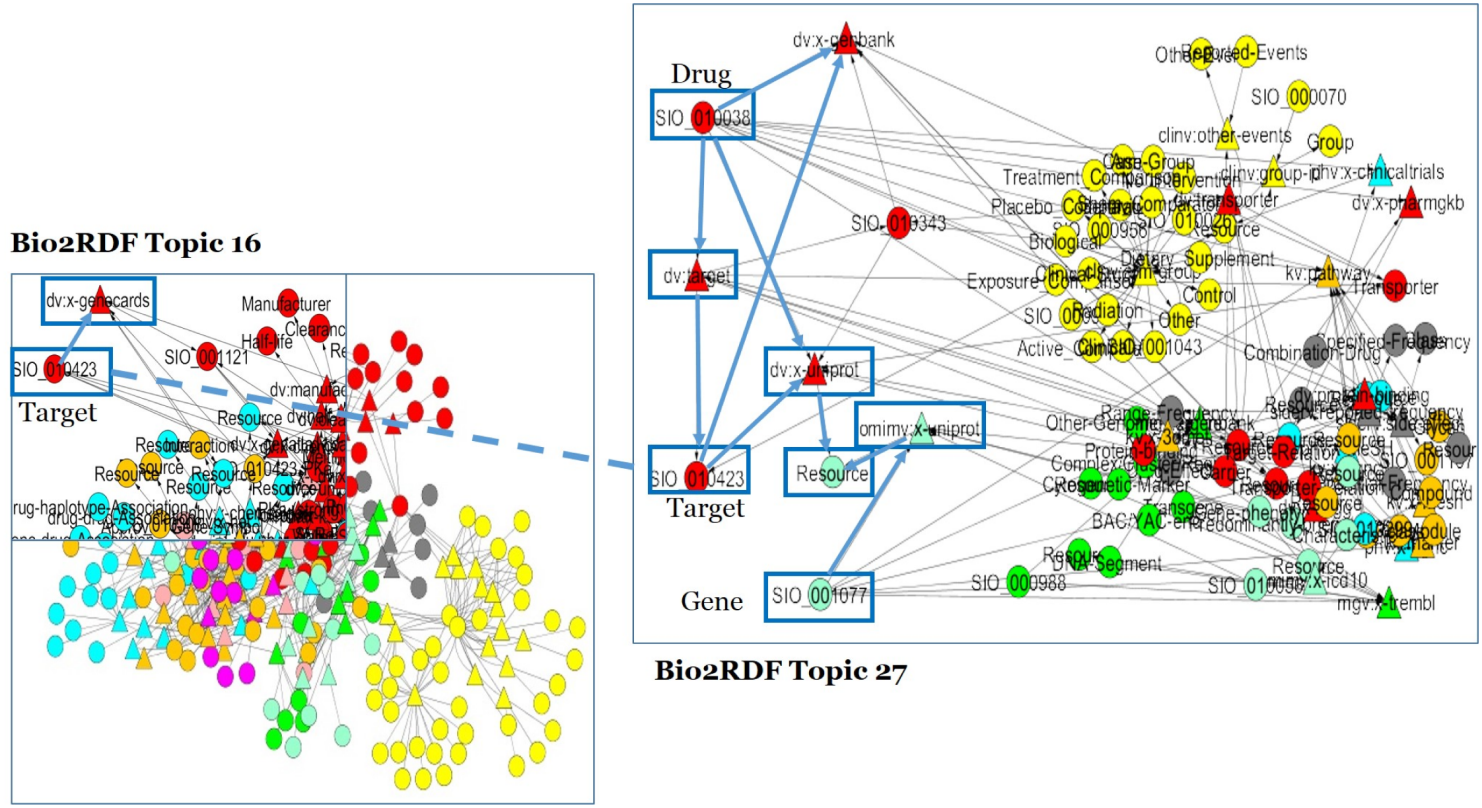
The Cross Domain Query Graphs for Topic 16 and Topic 27. In the cross domain query graph, the circle represents a concept and the triangle represents a predicate. A color is assigned to each domain as follows: DrugBank: Red; HGNC: Pink; MGI: Green; PharmGKB: Cyan; ClinicalTrials: Yellow; OMIM: Sky Blue; SIDER: Gray; KEGG: Orange; CTD: Magenta. The cross domain query graph is composed with the paths between Topic 27 and Topic 16 such as i) {*dv:Drug (SIO_010038)* → *dv:target* → *dv:Target(SIO_010423)* → *dv:x-genecards* → *dv:Gene(SIO_001121)*}; ii){*DrugBank*:〈*dv:Drug (SIO_010038)* → *dv:target* → *dv:Target(SIO_010423)* → *dv:x-uniprot*〉 ⇒ OMIM:*omimv:Uniprot*}; iii) {*omimv:Resource* → *omimv:x-uniprot* → *omimv:Uniprot*}.

In MedKDD, among 43 topics discovered from the Bio2RDF ontologies, there is a path between Topic 16 and Topic 27 through the common concepts such as *dv:Drug(SIO_010038)*, *dv:Target(SIO_010423)* and *dv:Gene(SIO_001121)* as shown in Fig 10. Specifically, the path includes *dv:Drug (SIO_010038)* → *dv:target* → *dv:Target(SIO_010423)* → *dv:x-genecards* → *dv:Gene(SIO_001121)* across these two topics. Table 6 shows the comparative analysis between the MedKDD and the SLAP frameworks in terms of the number of genes detected for top five drug instances. MedKDD could retrieve all the information for the given queries while SLAP retrieved either only partial information or no information at all. We have found that SLAP does not perform well in this experiment. It is because SLAP mainly focuses on the prediction on links between chemical compounds and targets with specific predicates including *bind*, *hasGo*, *hasSubstructure*, *hasPathway*, *hasTissue*, and *PPI*. Thus, some of information could not be retrieved from the query processing. However, the GraphKDD framework does not put any restriction on this query processing so that it has a capability to find any associations for a given query on drug, target, and gene.

**Fig 11:**
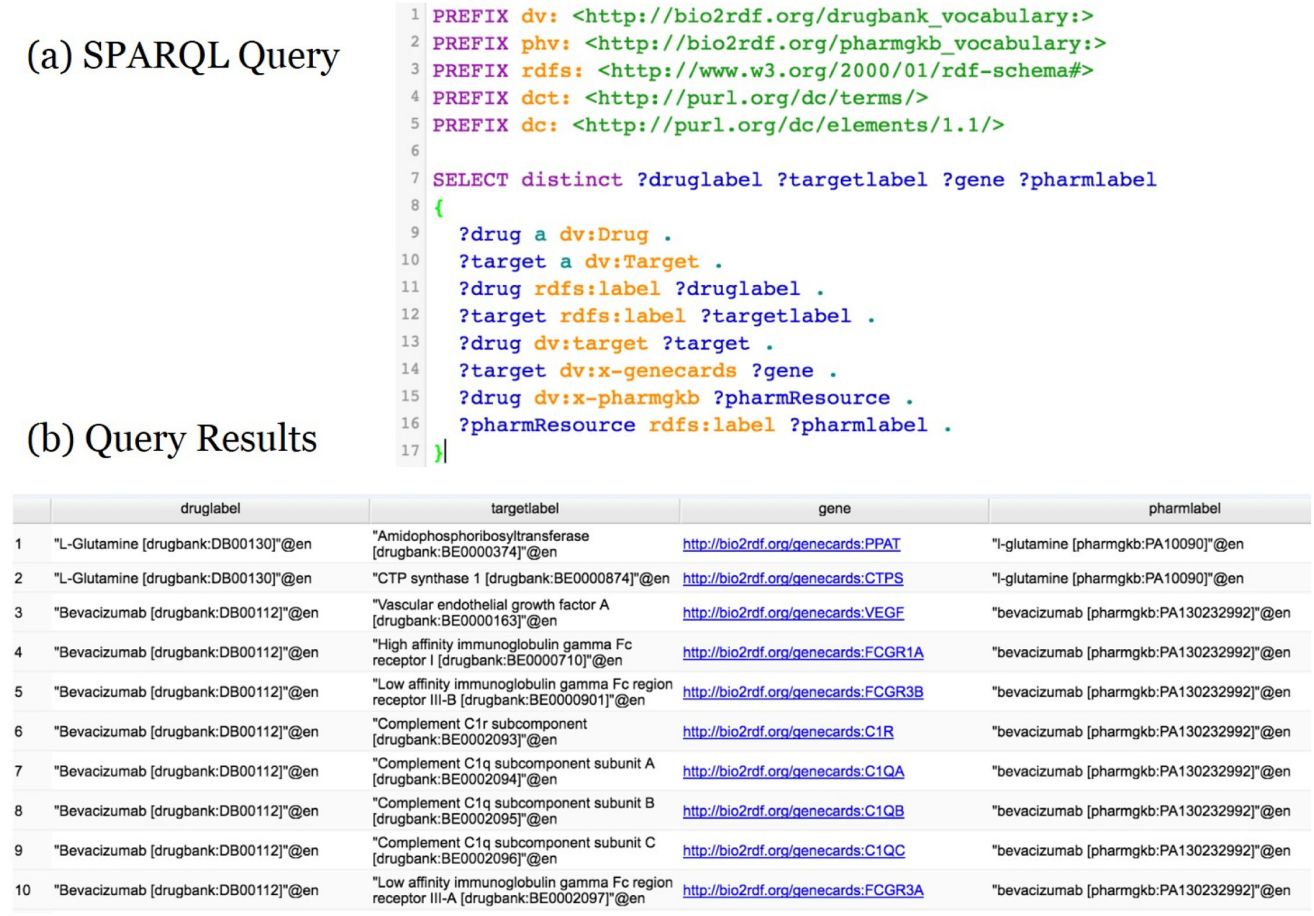
The Cross Domain SPARQL query and Query Results. The figure shows the SPARQL query (left) and query results for the cross domain query designed with the paths shown in Fig 10.

Second, for cross domain knowledge discovery, top five drug instances (i.e., *NADH*, *L-Glutamic Acid*, *Pyridoxal Phosphate*, *Ethanol*, and *Zonisamide*) were also selected according to the number of the instances associated with target, gene and *OMIM* resource. A cross domain query was designed with the following paths such as i) {*dv:Drug (SIO_010038)* → *dv:target* → *dv:Target(SIO_010423)* → *dv:x-genecards* → *dv:Gene(SIO_001121)*}; ii){*DrugBank*:〈*dv:Drug (SIO_010038)* → *dv:target* → *dv:Target(SIO_010423)* → *dv:x-uniprot*〉 ⇒ OMIM:*omimv:Uniprot*}; iii) {*omimv:Resource* → *omimv:x-uniprot* → *omimv:Uniprot*}. Table 7 shows the information retrieval comparison between the MedKDD and the SLAP frameworks in terms of the association with targets, genes, and *OMIM* resources for the top five drug instances. Fig 11 shows the SPARQL query and query results for the drug instances and their association with target, gene, *OMIM* resources. Similar to the first case, for the query across two domains *DrugBank* and *OMIM*, MedKDD retrieved all the relevant information while SLAP could not retrieve any information except partial information about drug and gene association.

**Table 7:** Top 5 Drug Instances & Cross Domain Query Results of MedKDD & SLAP.

Drug Name	MedKDD			SLAP		
	#Targets	#Genes	#Resources	#Targets	#Genes	#Resources
NADH [drugbank:DB00157]	143	141	204	0	0	0
L-Glutamic Acid [drugbank:DB00142]	62	62	90	0	94	0
Pyridoxal Phosphate [drugbank:DB00114]	58	54	73	0	56	0
Ethanol [drugbank:DB00898]	78	78	62	0	0	0
Zonisamide [drugbank:DB00909]	63	63	56	0	27	0

The results are from the cross domain query designed with the following paths such as i) {*dv:Drug (SIO_010038)* → *dv:target* → *dv:Target(SIO_010423)* → *dv:x-genecards* → *dv:Gene(SIO_001121)*}; ii){*DrugBank*:〈*dv:Drug (SIO_010038)* → *dv:target* → *dv:Target(SIO_010423)* → *dv:x-uniprot*〉 ⇒ OMIM:*omimv:Uniprot*}; iii) {*omimv:Resource* → *omimv:x-uniprot* → *omimv:Uniprot*}. The query about the drug and target association is from a single topic, Bio2RDF Topic 27. However, the query across two domains *DrugBank* and *OMIM*, MedKDD retrieved all the relevant information for this cross domain query while SLAP could not retrieve any information except partial information about drug and gene association.

In the comparative analysis, we demonstrated MedKDD’s capacity retrieving the association relationships between multiple concepts or predicates ether cross topics within a single domain or across domains. MedKDD shows the 100% accuracy rate in retrieving this information from the topics of nine different domains. Although SLAP proposed a strong statistical model to predict the association between drugs and genes, SLAP has a very limited capacity in retrieving information across topic (drug, target, and gene) or across domains (DrugBank and OMIM). The SLAP prediction of drug and gene interactions was strictly limited to the association between chemical compounds and targets. This result implicates the effectiveness of the MedKDD framework in discovering knowledge even across topics or across domains compared to the cross domain query processing approach, namely SLAP.

### Domain Collaboration Patterns in Cross Domains

**Fig 12:**
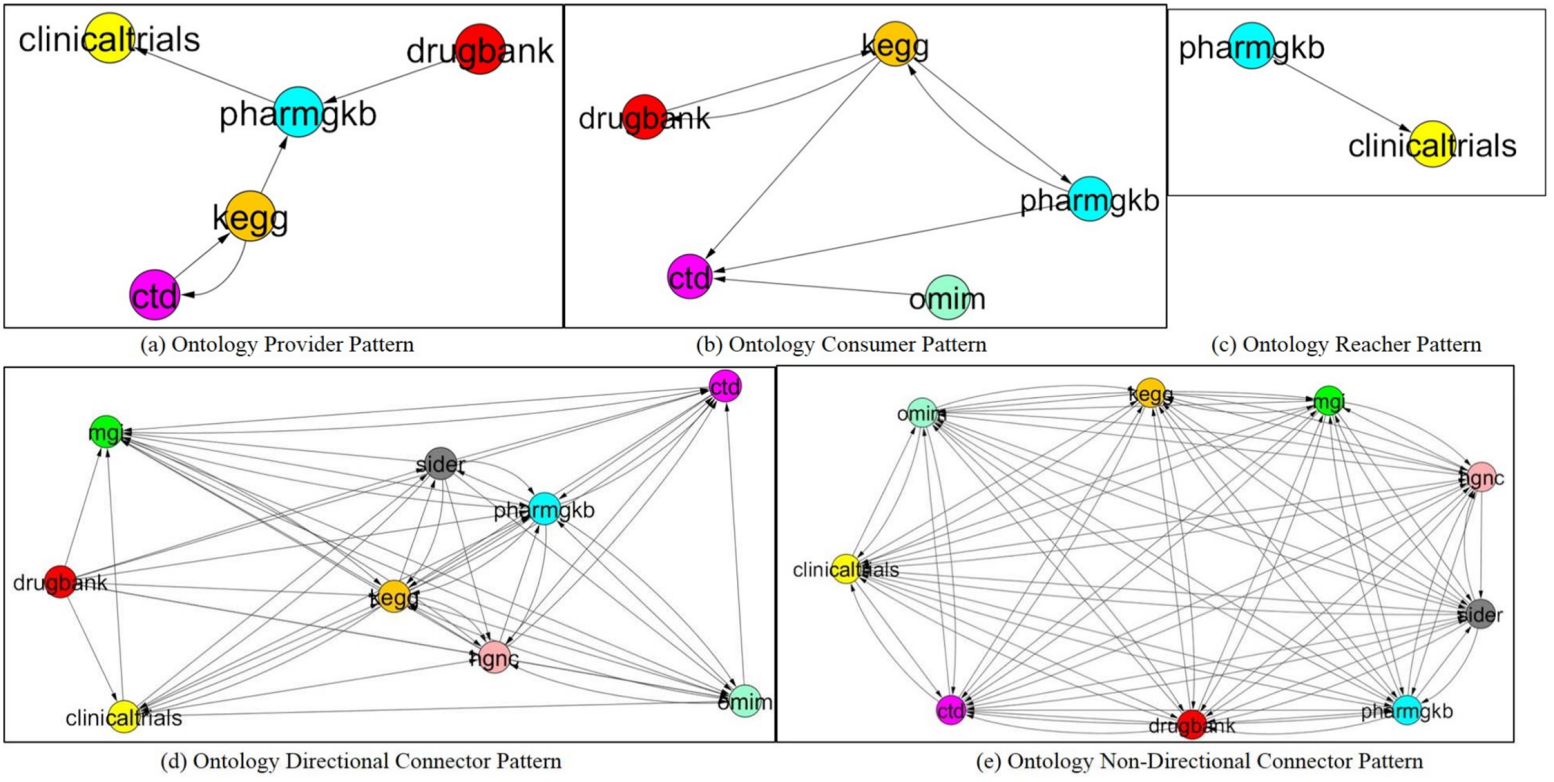
Cross Domain Pattern Graphs. (a) Topic 25: Provider Pattern Graph (b) Topic 15: Consumer Pattern Graph (c) Topic 22: Reacher Pattern Graph (d) Topic 16: DC Pattern Graph (e) Topic 23: NDC Pattern Graph.

**Fig 13:**
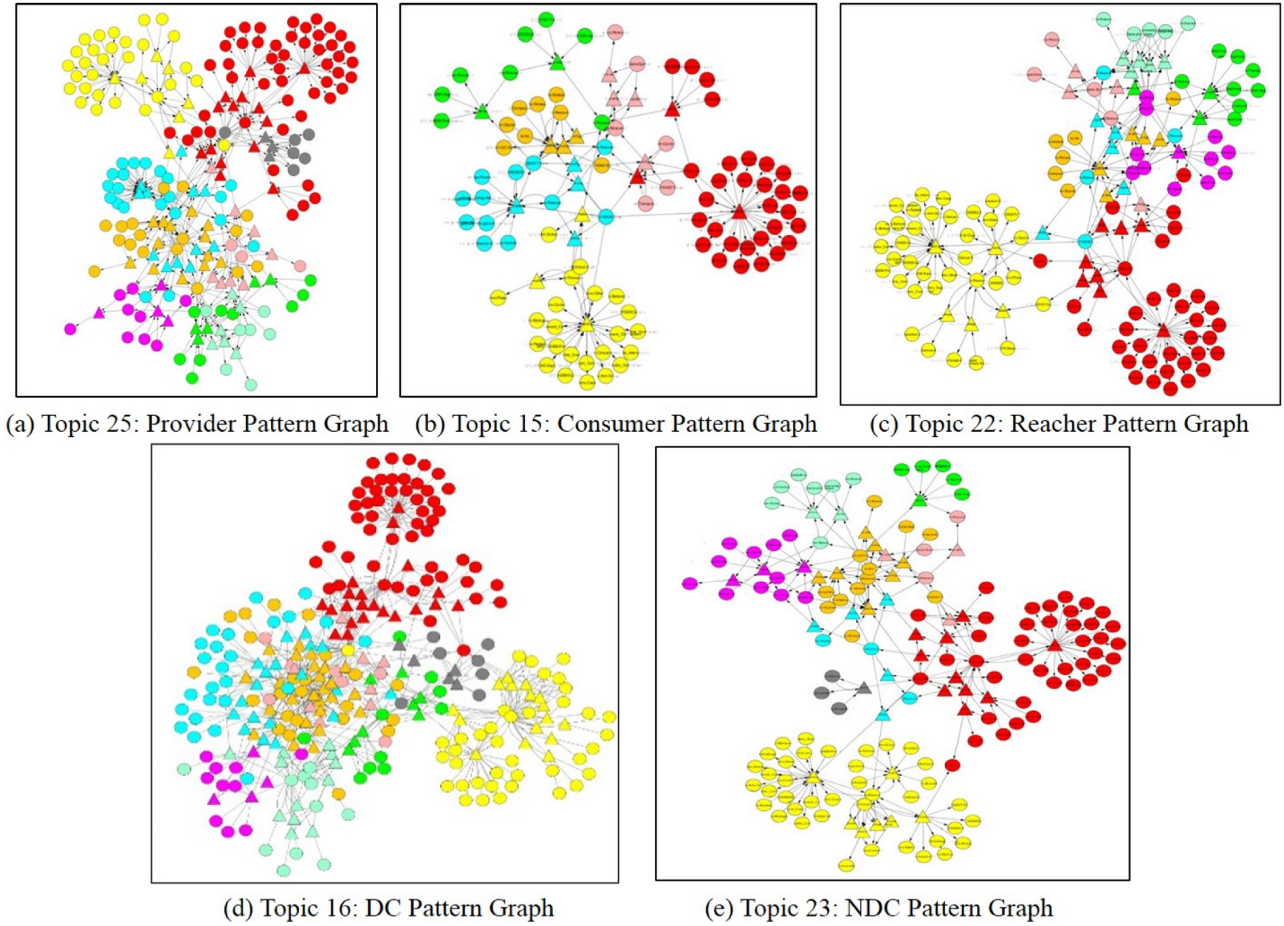
Cross Domain Collaboration Graphs. (a) Domain Provider Pattern (b) Domain Consumer Pattern (c) Domain Reacher Pattern (d) Domain Directional Connector Pattern (e) Domain Non-Directional Connector Pattern. A color is assigned to each domain as follows: DrugBank: Red; HGNC: Pink; MGI: Green; PharmGKB: Cyan; ClinicalTrials: Yellow; OMIM: Sky Blue; SIDER: Gray; KEGG: Orange; CTD: Magenta.

Based on top five CDNP patterns (Provider, Consumer, Reacher, Directional Connector, Non-Directional Connector), we analyzed the collaboration across domains as shown in Fig 12. Topic graphs are depicted in Fig 13. For each case study, we now show its topic pattern graph of concepts and predicates and the instances of concepts in this topic graph.

**Case 1:** *Provider* **Patterns in Domain Collaboration** Five domains (*DrugBank*, *PharmGKB*, *ClinicalTrials*, *KEGG* and *CTD*) are involved in the collaboration of the *Provider* pattern. In this collaboration, we found that *DrugBank* and *KEGG* are a *Provider*, *CTD* is a *Balancer*, and *PharmGKB* is a *Consumer* as well as a *Bridger*. *ClinicalTrials* is its *Consumer*. Fig 12(a) shows a domain collaboration graph for the given *Provider* pattern. Fig 13(a) shows the *provider* pattern graph of Topic 25.

Fig 1(a) shows a *Provider* pattern in Topic 25. This pattern describes the collaboration of two predicates, namely *phv:x-hgnc* and *kv:x-hgnc* to integrate information from three domains. Specifically, *PharmGKB* *Resource* links to *KEGG* *Gene* (SIO normalized) through *HGNC* *Gene symbol*. Table 8 shows 5 instances of the concepts in the *Provider* pattern of Topic 25.

**Table 8:** Provider Pattern in Topic 25.

phv:Resource	hv:Resource	SIO_001077:Gene
epidermal growth	factor receptor	Gene Symbol for EGFR EGFR, ERBB, ERBB1, HER1, PIG61, mENA; epidermal growth factor receptor (EC:2.7.10.1); K04361 epidermal growth factor receptor [EC:2.7.10.1]
complement component 1, r subcomponent	Gene Symbol for C1R	C1R; complement component 1, r subcomponent (EC:3.4.21.41); K01330 complement component 1, r subcomponent [EC:3.4.21.41]
complement component 1, q subcomponent, B chain	Gene Symbol for C1QB	C1QB; complement component 1, q subcomponent, B chain; K03987 complement C1q subcomponent subunit B
complement component 1, s subcomponent	Gene Symbol for C1S	C1S; complement component 1, s subcomponent (EC:3.4.21.42); K01331 complement component 1, s subcomponent [EC:3.4.21.42]
interleukin 2 receptor, beta	Gene Symbol for IL2RB	IL2RB, CD122, IL15RB, P70-75; interleukin 2 receptor, beta; K05069 interleukin 2 receptor beta

For the Provider pattern in Topic 25, the three important concepts and five instances are shown.

**Case 2: Domain Collaboration with** *Consumer* **Patterns** Five domains, namely *KEGG*, *OMIM*, *DrugBank*, *CTD*, and *PharmGKB*, are involved in this case. We found that *CTD* is a *Consumer* of *KEGG*, *OMIM* and *PharmGKB*. *DrugBank* are a *Balancer* with *KEGG*. Fig 12(b) shows a domain collaboration graph for the *Consumer* pattern, *CTD*. Fig 13(b) shows the *Consumer* pattern graph of Topic 15.

Fig 1(b) shows a *Consumer* pattern in Topic 15. This *Consumer* pattern shows the collaboration between predicates *mgv:x-ensembl-protein* and *kv:x-uniprot* as a *Consumer* of the *PharmGKB* concept (SIO normalized), *SIO_001077:Gene*. The collaboration is established across three domains such as *KEGG*, *MGI* and *PharmGKB*. In this pattern, due to the collaboration of these two *Consumer* predicates, the *Uniprot* concept *Resource* is linked to the *Ensemble* concept *Resource* through *PharmGKB* concept *Gene* (SIO normalized). Table 9 shows 5 instances of the concepts in the *Consumer* pattern in Topic 15.

**Table 9:** Consumer Pattern in Topic 15.

SIO_001077:Gene	ensev:Resource	unv:Resource
Brd7	ENSMUSP00000034085	Bromodomain-containing protein 7
C1qbp	ENSMUSP00000077612	Complement component 1 Q subcomponent-binding protein, mitochondrial
Ddx21	ENSMUSP00000042691	Nucleolar RNA helicase 2
Kcnab1	ENSMUSP00000047480	Voltage-gated potassium channel subunit beta-1
Nip7	ENSMUSP00000034392	60S ribosome subunit biogenesis protein NIP7 homolog

For the Consumer pattern in Topic 15, the three important concepts and five instances are shown.

**Case 3: Domain Collaboration with** *Reacher* **Patterns** Only two predicates from two domains, namely *PharmGKB* and *ClinicalTrials*, are involved in the *Reacher* pattern. From this pattern analysis, we found that *PharmGKB* plays a *Provider* and *ClinicalTrials* a *Consumer* from this collaboration. Fig 12(c) shows the domain collaboration with the *Reacher* pattern between *PharmGKB* and *ClinicalTrials*. Fig 13(c) shows the *Reacher* pattern graph of Topic 22.

Fig 1(c) shows the *Reacher* patterns in Topic 22. This *Reacher* pattern was formed with the predicates *kv:pathway* and *dv:x-kegg* across four domains (*PharmGKB*, *DrugBank*, *KEGG*, *CTD*). Through the collaboration of these two predicates in this pattern, the *PharmGKB* concept *Drug* (SIO normalized) is linked to the *KEGG* concept *Resource* and the *KEGG* concept *Resource* is linked to the *CTD* concept *Pathway* (SIO normalized). Table 10 shows 5 instances of the concepts in the *Reacher* pattern of Topic 22.

**Table 10:** Reacher Pattern in Topic 22.

SIO_010038:Drug	kv:Resource	SIO_001107:Pathway
L-Lysine	L-Lysine; Lysine acid; 2,6-Diaminohexanoic acid	ABC transporters
Succinic acid	Succinate; Succinic acid; Butanedionic acid; Ethylenesuccinic acid	Citrate cycle (TCA cycle)
Glycine	Glycine; Aminoacetic acid; Gly	Biosynthesis of amino acids
Pyruvic acid	Pyruvate; Pyruvic acid; 2-Oxopropanoate; 2-Oxopropanoic acid; Pyroracemic acid	Pentose phosphate pathway
L-Glutamic Acid	L-Glutamate; L-Glutamic acid; L-Glutaminic acid; Glutamate	Biosynthesis of secondary metabolites

For the Reacher pattern in Topic 22, the three important concepts and five instances are shown.

**Case 4: Domain Collaboration with** *Directional Connector* **Patterns** From the pattern analysis with top 40 predicates, all nine domains have the *Directional Connector* (DC) patterns. Fig 12(d) shows the domain collaboration through the *DC* patterns with 54 links among these domains. We have found that *CliniclalTrials*, *DrugBank* and *SIDER* play the role of *Provider* and *CTD*, *HGNC*, *KEGG*, *MGI*, *OMIM*, *PharmGKB* *Consumer*. Furthermore, *KEGG*, *PharmGKB*, *SIDER*, *HGNC* play the role of *Bridger*. The connection among the domains were established through the *Bridger* pattern. Fig 13(d) shows the *DC* pattern graph of Topic 16.

Fig 2(a) shows a DC pattern in Topic 16. In this *DC* pattern of Topic 16, three predicates such as *dv:x-hgnc*, *hv:x-omim* and *omimv:x-mgi* were used to connect concepts across five domains (*KEGG*, *DrugBank*, *HGNC*, *OMIM*, *MGI*). In this pattern, the *KEGG* concept *Enzyme* (SIO normalized) links to the *HGNC* concept *Resource*. The *HGNC* concept *Resource* links to the *OMIM* concept *Resource*, and the *OMIM* concept *Resource* links to the *MGI* concept *Resource*. We found all the paths within the bounded context (the maximum distance between predicates, *B* = 3) determined by the *DC* patterns. One of them is the path 〈*SIO_010343:Enzyme* → *dv:x-hgnc* → *hv:Resource* → *hv:x-omim* → *omimv:Resource* → *omimv:x-mgi* → *mgv:Resource*〉. Table 11 shows 5 instances of the concepts in the *DC* pattern of Topic 16.

**Table 11:** Directional Connector Pattern in Topic 16.

SIO_010343:Enzyme	hv:Resource	omimv:Resource	mgv:Resource
Prostaglandin G/H synthase 2	Gene Symbol for PTGS2	PROSTAGLANDIN-ENDOPEROXIDE SYNTHASE 2; PTGS2	Ptgs2
Vitamin K-dependent protein C	Gene Symbol for PROC	PROTEIN C; PROC	Proc
Cytochrome P450 2C9	Gene Symbol for CYP2C9	CYTOCHROME P450, SUBFAMILY IIC, POLYPEPTIDE 9; CYP2C9	Cyp2c65
CYP3A	Gene Symbol for CYP3A7	CYTOCHROME P450, SUBFAMILY IIIA, POLYPEPTIDE 7; CYP3A7	Cyp3a13
Cob(I)yrinic acid a,c-diamide adenosyltransferase, mitochondrial	Gene Symbol for MMAB	MMAB GENE; MMAB	Mmab

For the Directional Connector Pattern in Topic 16, the four important concepts and five instances are shown.

**Case 5: Domain Collaboration with** *Non-Directional Connector* **Patterns** In the *Non-Directional Connector* (NDC) pattern discovery, all the 9 domains are involved. Fig 12(e) shows the ontology collaboration through the *NDC* patterns. These 9 ontologies are connected with 72 links, which means all of them are fully connected. Interestingly, all of them have the same number of in-degree and out-degree, so that they are well balanced. Thus, no *Bridge* pattern is required in this collaboration. Fig 13(e) shows the *NDC* pattern graph of Topic 23.

Fig 2(b) shows a domain collaboration graph generated from the *NDC* pattern in Topic 23. This *NDC* pattern is composed with four predicates such as *mgv:x-refseq-transcript*, *ctdv:pathway* and *ctdv:disease* that are used to connect nine different domains (*KEGG*, *DrugBank*, *MGI*, *HGNC*, *SIDER*, *PharmGKB*, *ClinicalTrials*, *OMIM*, *CTD*). Specifically, in this pattern, those three predicates are used to connect six concepts such as *KEGG Gene* (SIO normalized), *Refseq resource*, *KEGG Resource*, *CTD Chemical*, *KEGG Pathway* (SIO normalized) and *CTD Chemical-disease-association*. Table 12 shows 5 instances of the *NDC* pattern in Topic 23.

**Table 12:** Non-Directional Connector Pattern in Topic 23.

SIO_001077 (Gene)	refv:Resource	v:Resource	Chemical-Disease-Association	Chemical	SIO_001107 (Pathway)
Fbxl12	NM_001002846	SDKD	1,10-phenanthroline (C025205) & Plasminogen Activator Inhibitor-1 Deficiency	Plasminogen Activator Inhibitor-1	p53 signaling pathway
Gjb6	NM_001010937	SDKD	2-nitro-4-phenylenediamine (C014706) & Interleukin 2 Receptor, Alpha, Deficiency of	Interleukin 2, Receptor Alpha	Cytokine-cytokine receptor interaction
Dclre1b	NM_001025312	SDKD	2-(methylamino)isobutyric acid (C017911) & Insulin-Like Growth Factor I Deficiency	Insulin-Like Growth Factor I	Oocyte meiosis
BC053393	NM_001025435	SDKD	2-methoxy-5-(2’,3’,4’-trimethoxyphenyl) tropone (C030370) & Combined Saposin Deficiency	Combined Saposin	Lysosome
Maf	NM_001025577	SDKD	2-methoxy-5-(2’,3’,4’-trimethoxyphenyl) tropone (C030370) & Krabbe Disease, Atypical	Combined Saposin	Metabolism

For the Non-Directional Connector Pattern in Topic 23, the six important concepts and five instances are shown. in the table, SDKD is Synthesis and degradation of ketone bodies refseq:NM_001002846, Chemical describes the deficiency of chemical components.

**Fig 14:**
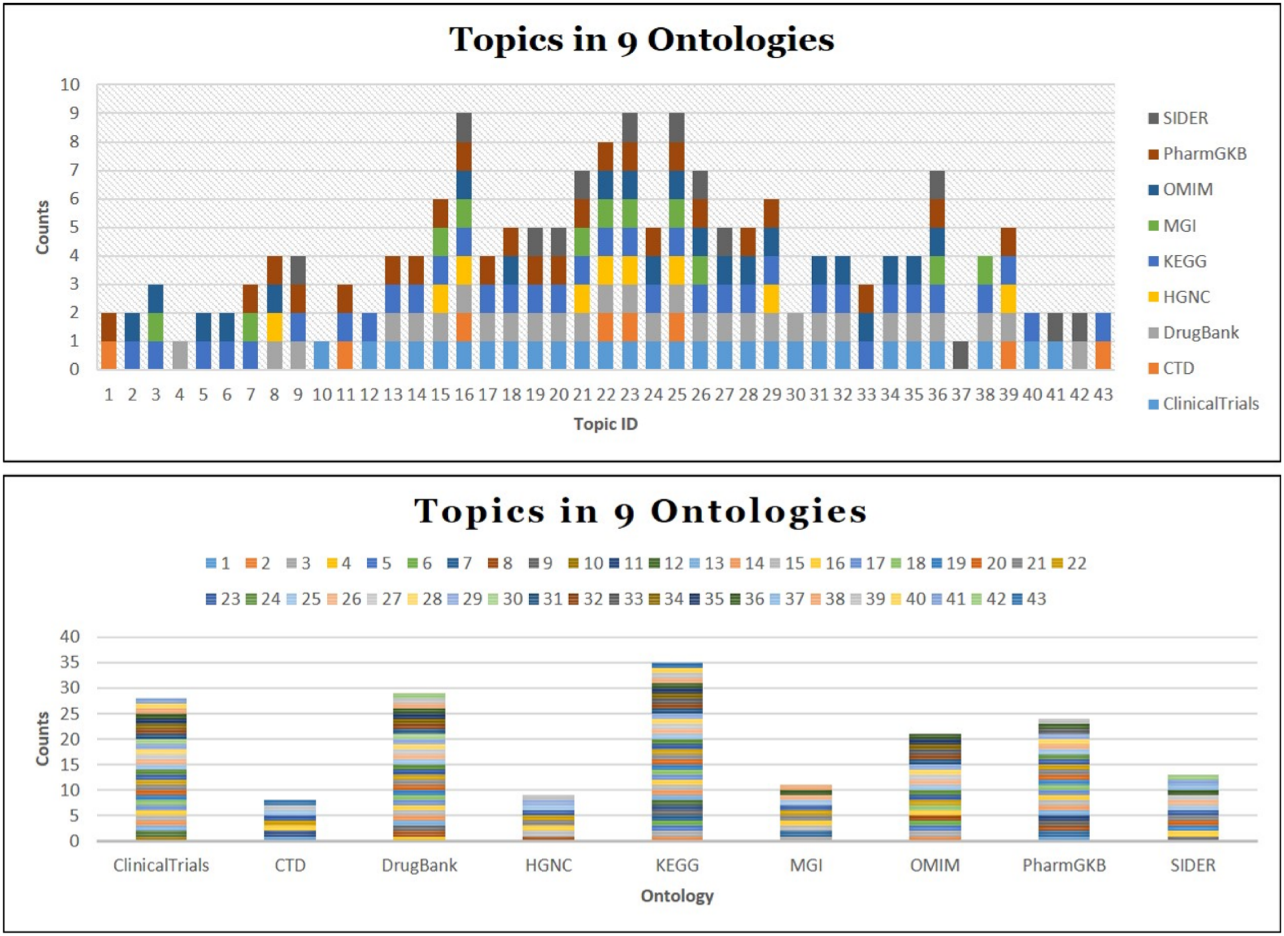
43 Topics Discovered from 9 Medical Domains. 40 Cross Domain Topics and 3 Single Domain Topics.

**Case 6: Domain Collaboration with Topics** The 43 topics discovered from 9 domains are shown in Fig 14. First, we present how the 43 topics are composed with the concepts and predicates from these domains. Topic 16, Topic 23, Topic 25 are the most diverse topics whose concepts and predicates are from all 9 different domains. On the other hand, Topic 4, Topic 10 and Topic 17 are from a single domain, *DrugBank*, *MGI*, *SIDER*, respectively. Second, the number of topics that were discovered from *KEGG*, *DrugBank*, *ClinicalTrials* is 35, 29, 28, respectively. They are three highest topic numbers among 9 domains. On the other hand, *CTD* and *HGNC* contain less than 10 topics, which are two smallest ones. In terms of the size of these domains, *ClinicalTrials*, *DrugBank* and *KEGG* are the top three biggest ones while *HGNC* and *CTD* are the bottom two smallest ones. Thus, we found that the size of domains (specially, the number of predicates) is strongly related to the number of topics in these domains.

## Discussion

### Knowledge Discovery with Heterogeneous Medical Data

There are many efforts that have been made for semantic annotation of heterogeneous data and perform knowledge discovery on biomedical data [34–37]. Most of these work have mainly focused on building or using ontologies for data normalization, connecting, and reasoning. Chen et al. [34] annotated different domains into a single ontology and provided an approach to find existing links between existing sources and targets as well as predict missing links between potential sources and targets. Data normalization and data integration platforms have been built for single domain and cross domain knowledge discovery. For the purpose, some medical ontologies are introduced, namely Bio2RDF (Linked Data for the Life Sciences) [6], TMO (Translational Medicine Ontology) [38], Chem2Bio2RDF (Linked Open Data Portal for Chemical Biology) [39], SIO (Semanticscience Integrated Ontology) [40], ATC (Anatomical Therapeutic Chemical) and DrugBank [41], Chem2Bio2OWL(Ontology for Chemogenomics/Systems Chemical Biology) [42], LLD (Linked Life Data) [43], LODD (Linked open drug data) [44] and LinkedCT (A Linked Data Space for Clinical Trials) [45].

We now discuss existing work on knowledge discovery. For relation extraction, ontologies are helpful for extraction of relations in the form of thesaurus, dictionary, or general corpus [46], for extraction of semantic knowledge of relations based on Metathesaurus and Semantic Network of UMLS [47], and for semantic search indexes [48]. Semantic rules have been applied to extract relations from publications [49]. Relations can also be extracted based on specific patterns such as protein-to-protein relations [50], gene-disorder association [51], and diseases and drugs [52]. Shotton et al. [53] presented semantic enhancement methods through citation context and semantically relations for biomedical research articles on tropical diseases.

A variety of research have been conducted in systematical and computational knowledge discovery with cross domain datasets. HeteSim is a general framework that was designed for relationship discovery and linking detection from heterogeneous networks [54, 55]. The iPHACE framework was designed to extract knowledge between drug-target interaction [56]. ChemProt [57] provided a database to discover relationships between disease and chemical biology. STITCH 3 [58] performed knowledge discovery between chemicals and proteins. Oprea et al. [59] built an integrated platform of drugs, targets, and clinical outcomes for supporting Drug repositioning. Kinnings et al. [60] discovered relationship between drug and disease by deploying chemical and systems biology. In [61] an ontology of chemical information entities was developed for the integration of calculated properties of chemical entities within a semantic web context. Campillos et al. [62] identified drug target by using side-effect similarity and then found the association among drug, target, and side effect. Connectivity Map [63] was designed to use gene-expression signatures in discovery of relationships among small molecules, disease, gene, and drug.

However, our approach is different in that, first of all, we focus on a more general approach for graph structural pattern analysis and topic discovery from heterogeneous information networks. In addition, we have combined an unsupervised learning algorithm with a pattern discovery technique to provide a more systematic way of knowledge discovery from multiple domains.

### Ontology Mapping and Alignment

Our approach for finding roles in ontology collaboration is related to existing work in ontology matching, alignment, classification and mapping [27]. Ontology mappings and aliments are essential in advanced semantic searchesand reasoning over integrated ontologies [64]. Recent work on ontology alignment have emphasized the importance of attributes in mapping between source and target concepts as well as the role played by the neighborhood of a concept [65, 66]. Specifically, [65] are interested in the identification of evolving mapping among multiple ontologies, characterizing their evolution as well as facilitating the impacted mappings. Similarity measures were defined for identification of relevant attributes for the mappings [66]. A semantic analysis for understanding the meaning of data has been achieved through mappings and alignments in biomedical systems [67]. The proposed approach in this paper would be effective in the analysis of collaboration between ontologies and their roles. This analysis will be useful to identify potential candidates for mappings and alignments that guarantee a consistent integration of models and interoperability for biomedical applications.

### Pattern-based Analysis

Pattern based knowledge analysis has been conducted in many aspects of biomedical research. van Leeuwen [68] proposed an interactive way to mine data by applying pattern-based mining method. Warrender and Lord [69] proposed an axiom based pattern driven approach in biomedical ontology engineering. Wang et al. [70] designed a biomedical pattern discovery algorithm based on a supervised learning approach. Rafiq et al. [71] developed an algorithm to discover temporal patterns in genomic databases. In [72], Gotz presented a method for data mining and visual analysis on clinical event patterns using electronic health record data. WHIDE was proposed for co-location pattern mining in multivariate bioimages [73]. Huang et al. [74] presented a clinical pathway pattern discovery method by using probabilistic topic models. Lasko et al. [75] proposed an unsupervised learning method for computational phenotype pattern discovery using clinical data. These works were different from ours because the discovered patterns in our approach were further analyzed for transforming to topics by clustering and ranking, and then represented in a hierarchical manner.

Our work is motivated by previous work that emphasised the importance of ontological relations. Tartir et al. [76] pointed out that there are numerous meaningful relations other than class-subclass relations that would be useful for understanding the ontologies. Shi et al. [77] provided a predicate oriented path finding approach by analyzing facts in large knowledge graphs. VEPathCluster [78] proposed a combination of vertex-centric and edge-centric approach for meta path graph analysis for enhancement of clustering quality of cross domain datasets. Sabou et al. [79] considered ontological relations to be the primary criterion for the summary extraction of ontologies, in which a relatively small number of concepts typically have a high degree of connectivity through hops. Pesquita et al. [80] proposed classification according diverse strategies suing different semantic similarity measures such as node-based/edge-based and pair-wise/group-wise.

In our study, we hypothesize that an association measurement based on predicate neighborhood patterns would be more effective in finding relevant information than a concept-based measurement. Our approach defined a new model of predicate-based patterns and neighboring closeness for an automatic knowledge discovery. In this paper, we fully focus on the discovery of cross domain patterns from the heterogeneous information network representing different types of objects and links in multiple biological ontologies. The MedKDD framework was designed to effectively discover topics from multiple ontologies by partition them into smaller topic graphs and constructing a topic hierarchy. The topic hierarchy was constructed based on the analysis of the discovered patterns and participating graphs into smaller sub-graphs. To our knowledge, there is no existing work that aim to discover cross domain topics based on predicate-oriented neighborhoods patterns discovered from multiple ontologies and use the discovered topics for knowledge discovery across domains.

## Conclusion

In this paper, we presented the MedKDD framework for knowledge discovery and semantic interoperability through the discovery of the Cross Domain Neighborhood Patterns (CDNP) from the heterogeneous information network of the multiple medical ontologies. In MedKDD, we developed the bottom-up hierarchical clustering (HPAL) algorithm and discovered cross domain topics from the given multiple ontologies. We demonstrated that cross domain cohesive topics can be dynamically discovered from heterogeneous information networks of multiple ontologies and used for cross domain knowledge discovery. The MedKDD framework was evaluated using a case study with nine ontologies of Bio2RDF and compared with the cross domain query processing approach, namely SLAP. Overall, the experimental results confirm that the MedKDD framework is effective in the cross domain knowledge discovery from heterogeneous information networks of multiple ontologies.

Future work will include the development of Apache Spark framework that is an extension of Hadoop for parallel and distributed knowledge discovery processing from heterogeneous information network [81]. For the assertion retrieval and clustering, we will explore existing parallel and distributed approaches such as the NIMBLE project [82], Apache Mahout library, and the Distributed Co-clustering (DisCo) framework [83] that have been used successfully in diverse applications for extremely large datasets.
